# Comparative study of immune response to local tumor destruction modalities in a murine breast cancer model

**DOI:** 10.3389/fonc.2024.1405486

**Published:** 2024-06-18

**Authors:** Sadna Budhu, Kwanghee Kim, Wesley Yip, Stephen La Rosa, Sylvia Jebiwott, Liqun Cai, Aliya Holland, Jasmine Thomas, Dina Preise, Alex Somma, Benjamin Gordon, Avigdor Scherz, Jedd D. Wolchok, Joseph Erinjeri, Taha Merghoub, Jonathan A. Coleman

**Affiliations:** ^1^ Swim Across America and Ludwig Collaborative Laboratory, Department of Pharmacology, Weill Cornell Medical Center, New York, NY, United States; ^2^ Department of Surgery, Memorial Sloan Kettering Cancer Center, New York, NY, United States; ^3^ Urology Service, Department of Surgery, Memorial Sloan Kettering Cancer Center, New York, NY, United States; ^4^ Department of Radiology, Memorial Sloan Kettering Cancer Center, New York, NY, United States; ^5^ Department of Plants and Environmental Sciences, The Weizmann Institute of Science, Rehovot, Israel; ^6^ Department of Immunology, Weill Cornell Medical Center, New York, NY, United States; ^7^ Department of Medicine, Parker Institute for Cancer Immunotherapy and Sandra and Edward Meyer Cancer Center, Weill Cornell Medical Center, New York, NY, United States

**Keywords:** radiation therapy, vascular photodynamic therapy, cryoablation, immune checkpoint blockade, breast cancer

## Abstract

**Introduction:**

Immunotherapy is revolutionizing the management of multiple cancer types. However, only a subset of patients responds to immunotherapy. One mechanism of resistance is the absence of immune infiltrates within the tumor. *In situ* vaccine with local means of tumor destruction that can induce immunogenic cell death have been shown to enhance tumor T cell infiltration and increase efficacy of immune checkpoint blockade.

**Methods:**

Here, we compare three different forms of localize tumor destruction therapies: radiation therapy (RT), vascular targeted photodynamic therapy (VTP) and cryoablation (Cryo), which are known to induce immunogenic cell death, with their ability to induce local and systemic immune responses in a mouse 4T1 breast cancer model. The effects of combining RT, VTP, Cryo with anti-PD1 was also assessed.

**Results:**

We observed that RT, VTP and Cryo significantly delayed tumor growth and extended overall survival. In addition, they also induced regression of non-treated distant tumors in a bilateral model suggesting a systemic immune response. Flow cytometry showed that VTP and Cryo are associated with a reduction in CD11b+ myeloid cells (granulocytes, monocytes, and macrophages) in tumor and periphery. An increase in CD8+ T cell infiltration into tumors was observed only in the RT group. VTP and Cryo were associated with an increase in CD4+ and CD8+ cells in the periphery.

**Conclusion:**

These data suggest that cell death induced by VTP and Cryo elicit similar immune responses that differ from local RT.

## Introduction

Immunotherapies have shown great potential to control cancer by enhancing T cell responses to cancer antigens. Immune checkpoint blockade (ICB) targeting the cytotoxic T-lymphocyte associated protein 4 (CTLA-4), programmed cell death protein 1 (PD-1) and lymphocyte activating gene 3 (LAG-3) have been approved for treating multiple cancers, however, a large fraction of patients still do not respond to these therapies. This is mainly due to multiple mechanisms of immune resistance that may exist within tumors, such as the lack of T cell infiltration or immune desert tumor microenvironment (TME), low tumor mutation burden or an immunosuppressive TME. In poorly infiltrated tumors (cold tumors), enhancing tumor T cell infiltration by physically disrupting the tumor can be utilized using local ablation therapies, such as with radiation therapy (RT), vascular targeted photodynamic therapy (VTP), or cryoablation (Cryo). In highly infiltrated tumors (hot tumors), immune suppressive cells have been shown to play a major role in limiting the efficacy of anti-tumor immunity (1). The presence of high levels of immune suppressive cells such as myeloid derived suppressive cells (MDSCs) have been shown to correlate with poor prognosis and ICB resistance (2, 3). Combination strategies that can convert a cold tumor into a hot tumor while targeting the immunosuppressive TME are key to the design of therapeutic combinations that can overcome such resistance mechanisms.

Local ablative therapies have all been shown induce tumor cell death that can be immunogenic (4–6) because they not only release tumor antigens that can trigger an *in-situ* vaccination effect but also secreted factors such as danger associated molecular patterns (DAMPs) that can facilitate the maturation of antigen presenting cells (APCs) further enhancing the immune response to the cancer (7). In addition, inflammatory or secreted factors such as cytokines can recruit more immune cells into to the tumor microenvironment. The resulting outcome of immunogenic cell death in the context of cancer is activation of a tumor specific immune response in the host. Therefore, not only can the immune system recognize and kill the primary tumor but also distant metastases (termed the abscopal effect).

Prior studies have demonstrated that the abscopal effect of RT, in which localized RT causes distant tumors to regress, may be due to the release of tumor-antigens that can enhance the immunogenicity of tumors, which can then increase susceptibility to systemic immune-stimulating agents (8). This effect has been seen in the regression of a metastatic melanoma case treated with local RT, which enhanced systemic responses to an anti-CTLA-4 antibody (ipilimumab) (9). This co-occurred with increase in NY-ESO-1 expression and CD4^+^ICOS^high^ cells, both of which correlated with increased benefit from ipilimumab. In a recent clinical study of RT in combination with CTLA-4 blockade in chemo-refractory metastatic non-small-cell lung cancer (NSCLC) where anti-CTLA-4 antibodies had failed to demonstrate significant efficacy alone or in combination with chemotherapy, RT and CTLA-4 blockade showed objective responses in 18% patients and 31% disease control and induced systemic anti-tumor T cells (10). In preclinical breast cancer models, radiation controls tumor growth both directly through cell killing and indirectly through immune activation (11).

Another local therapy with both ablative and immunologic effects is VTP, a form of photodynamic therapy, which uses an intravenously administered photosensitizing agent (padeliporfin/TOOKAD Soluble/WST11; STEBA Biotech, Luxembourg) and near-infrared light to create radical oxygen species that lead to tumor vasculature collapse and subsequent tissue destruction (12). Photodynamic therapy, which is Food and Drug Administration-approved for several malignancies (13), has been reported to cause acute inflammation and increase tumor antigen presentation (14). Similarly, VTP treatment of preclinical models induces a defined local inflammatory response, including IFNγ production and infiltration of tumors by T cells and neutrophils in colon cancer models (15). In an orthotopic murine model of renal cell carcinoma that develops lung metastases, VTP in combination with anti-PD-1/PD-L-1 antibodies demonstrates superior anti-tumor activity as compared to checkpoint blockade alone and induces immune infiltration in primary and metastatic sites (16). Modulation of PD-L1 expression by VTP in human xenograft tumors was also observed.

An additional local ablation option is Cryo, which employs needle applicators (cryoprobes) to transmit pressurized argon and helium gases to the tumor to cause localized tissue freezing and thawing (17). As intracellular and extracellular fluid freezes, tissue destruction results from cell membrane disruption by ice crystals, cellular dehydration, and vascular thrombosis at temperatures below -20 to -40°C. Cryo induces cell lysis and leaves tumor proteins and tumor-associated antigens immunologically intact, different from hyperthermia-based ablation modalities (18). Cryo also results in the induction of both a tumor-specific T cell response in the tumor draining lymph node (TDLN) and increased systemic NK cell activity, correlating with rejection of tumors upon re-challenge in a murine model of breast cancer (18). When performed in conjunction with CTLA-4 blockade, Cryo improved survival in a TRAMP C2 mouse model of prostate cancer, also generating intratumoral and systemic expansion of CD8^+^ T cells against the SPAS-1 tumor-specific antigen (19). A safety/feasibility study of preoperative single-dose ipilimumab and/or Cryo in 19 women with early-stage breast cancer showed that this combination strategy increased Th1-cytokine production, peripheral T cell proliferation/activation, and intratumoral proliferation of effector T cells relative to regulatory T cells (20).

Since RT, VTP and Cryo are all clinically available treatment options for overlapping but also different indications, it is imperative to understand the immune modulatory effect of each therapy to delineate the best strategy in combination with immunotherapy. As each of these modes of treatment are given locally to the tumors only and have been shown to synergize well with immune modulatory agents, we compared the effects of three local ablation therapies: RT, Cryo and VTP using 4T1 triple negative murine breast cancer (TNBC) model. Unlike melanomas such as mouse B16 melanoma, 4T1 tumors have been shown to be poorly infiltrated by T cells but highly infiltrated with myeloid (CD11b^+^) immune cells (2). These CD11b^+^ myeloid cells in the spleens and tumors of 4T1 bearing mice display a MDSC phenotype and have been shown to be functionally immunosuppressive (2, 3). Thus, 4T1 tumors represent the classical T cell desert or immunologically “cold” tumors but highly immunosuppressive (enriched in MDSCs) that have been shown to contribute to the poor response of 4T1 tumors to ICB.

The main goal of this study is to measure how RT, VTP, or Cryo effect the innate and adaptive immune responses not only in the treated tumors but also on the immune system systemically. Our results demonstrate that immune modulation with RT, VTP, or Cryo therapy can generate potent local and systemic antitumor responses. These modalities may represent an effective strategy to treat poorly infiltrated or immunologically “cold” tumors known to be resistant to ICB by modulating the immunosuppressive TME along with systemic T cell activation to enhance therapeutic efficacy of ICB.

## Materials and methods

### Cell line

The murine breast cancer cell line 4T1 was maintained in RPMI medium supplemented with 10% FBS, 2 mM L-glutamine and penicillin with streptomycin.

### *In vivo* studies

2x10^5^ 4T1 cells were subcutaneously injected in the right hindlimb of BALB/cJ female mice (7–8 weeks old, JACKSON LABORATORY, Bar Harbor, ME) 12 days prior to RT, VTP and Cryo. For bilateral models, two sequential injections of 2x10^5^ 4T1 cells 12 days and 5 days prior to the treatments were administered in the mice. The mice bearing tumors were randomly assigned to different cohorts for further experiments. For efficacy studies (tumor growth and survival), n = 10 mice were used for each experimental group (Control, RT, VTP, Cryo +/- anti-PD1) and for *ex vivo* studies (e.g., flow cytometry, IHC, cytokines), n = 5 mice were used for each experimental group. Local tumor growth was monitored twice a week using caliper measurements (Perkin-Elmer, Waltham, MA). The tumor area/volume curves averaged at each time point per group are reported for as long as all mice in a group are alive. Overall survival curves of each treatment group were analyzed using the Kaplan–Meier estimator. The survival curves measure time from start of experiment to a tumor size of 2000 mm^3^ (in which case the mouse needs to be euthanized) or death.

### Tumor directed radiation therapy

A single dose of 15 Gy was used to irradiate 4T1 tumors. This dose was previously determined to be optimal for immune infiltration in a mouse B16 melanoma model (21). On day 12, mice were anesthetized with isoflurane and placed in a radiation jig where only the hindlimb is exposed (22). The right hindlimb bearing 4T1 tumors was irradiated with 15 Gy RT using the X-RAD 320 focus beam irradiator such that only the exposed hindlimb bearing the tumors receives the radiation.

### WST-11 VTP

WST-11, a photosensitizer, was reconstituted in sterile 5% dextran in water at 2 mg/mL under light protected condition and the aliquots were stored at -20°C. At the time of VTP treatment, an aliquot was thawed and filtered through 0.2 μm disc syringe filter (Sartorius Stedin Biotech North America, Bohemia, NY). The mice were intravenously infused with WST-11 (9 mg/kg) for 5 min followed immediately by 10 minutes laser (Modulight, Tampere, Finland) illumination (755 nm, 100 mW/cm) through a 1 mm frontal diffuser fiber (MedLight S.A., Ecublens, Switzerland). The light field was set up to cover the entire tumor area plus 1 mm rim using red-light aiming beam.

### Cryoablation

Visually guided cryoablation with 1.7 mm Per Cryo probes was used to treat tumors. Mice were anesthetized with a mixture of 100 mg/kg ketamine and 10 mg/kg xylazine, and the targeted area were shaved and cleaned with iodine and 70% alcohol swabs. A small cut in the skin was made to allow the cryoprobe tip to enter the tumor. Freeze cycle was discontinued when ice crystals encompassed the visible tumor and thaw cycles was discontinued when ice crystals were no longer visible. The treatment parameter was evaluated using single cycle at 20% freeze rate for up to 60 seconds. A control group was not ablated. After the treatment, mice were monitored for bleeding or other complications according to the standard recovery procedure of the approved animal protocol.

### Dosing schedule of anti-PD1

Animals received three doses of intraperitoneal (i.p.) injection of anti-PD1 (BioXCell, West Lebanon, NH; clone RMP1–14, 250 ug/mouse) on the same day of and 3- and 6-days post treatment.

### Immunohistochemistry

Tumors and spleens from 3 mice/group were harvested 6 days after treatment with RT, VTP and Cryo. Tissues were then fixed in 10% buffered formalin (Fisher Scientific, Pittsburgh, PA), embedded in paraffin, sectioned at 5 micrometer thickness, and stained with hematoxylin-eosin (H&E), anti-mouse CD8 and CD11b antibodies. Slides were scanned and analyzed for immunoreactivity the image analysis platform HALO by Indica Labs (Albuquerque, NM). For quantification, heathy areas within tumors and spleens were selected in parallel sections based on H&E staining. 3–5 regions/slide were selected and the percent of CD8^+^ and CD11b^+^ area were calculated and normalized to tissue surface area.

### Isolation of tumor-infiltrating cells and leukocytes and flow analysis

Spleens, LNs and tumors were prepared by mechanical dissociation through 40 and 100 μm cell strainers (BD Biosciences, Franklin Lakes, NJ) in RPMI supplemented with 7.5% FCS to isolate single cells. Red blood cells (RBCs) were lysed with ACK Lysing Buffer (Lonza, Basel, Switzerland) when required, all samples were washed and resuspended in FACS buffer (PBS without Ca^++^/Mg^++^ supplemented with 0.5% BSA and 2mM EDTA). For staining for flow cytometry analysis, 100–200μL of single cell suspensions of each tissue were plated into two 96-well round bottom plates: one plate for T cell analysis (anti-CD45-Alexa 700, anti-CD8-PerCP-Cy5, anti-CD4-v450, anti-CD25-APC-Cy7, anti-CD62L-PE, anti-CD44-PE-Cy7, anti-FoxP3-APC, anti-Ki67-FITC, anti-Granzyme B PE-Texas Red, and a fixable viability dye eFluor506 (Invitrogen, Waltham, MA)) and one plate for myeloid cell analysis (anti-CD45-FITC, anti-CD11c-PE, anti-CD8-PE-Texas Red, anti-Ly6G-PerCP-Cy5, anti-Ly6C-PE-Cy7, anti-MHCII-v450, anti-CD86-APC, anti-CD11b-APC-Cy7, and the viability dye eFluor506). Cells were pelleted by spinning at 2,000 rpm for 5 mins. Cells were incubated in 100μL of 5 μg/ml Fc-block antibody (clone 2.4G2, BD Biosciences) for 20 mins on ice in FACS buffer. After Fc-block, cells were stained in FACS buffer containing fluorophore conjugated surface antibodies and for 20 mins on ice. Samples were then washed 2 times with 200μL FACS buffer. All intracellular staining were done using the Foxp3 fixation/permeabilization buffer according to the manufacturer’s instructions. Events were acquired using a multi-channel flow cytometer (BD LSRII, BD Biosciences, San Diego, CA). FlowJo software V10 (FlowJo,LLC) was used to analyze all data. The gating strategies for each myeloid and T cell panel are shown in **Supplementary Figures S2**, **S6**. We identified the immune cell populations as follows: live cells – viability dye negative, total immune infiltrates CD45^+^, total myeloid cells – CD45^+^CD11b^+^. Myeloid cells were subgated into Neutrophils/Granulocytes – Ly6G^+^, Monocytes – Ly6C^+^, Macrophages – Ly6C^-^Ly6G^-^. Antigen presenting cells (APCs/DCs) were identified as CD45^+^CD11c^+^MHC II^+^ and subgated into cross-presenting DCs (CD8^+^ DCs) – CD8^+^, conventional DCs (CD11b^+^ DCs) –CD11b^+^, other DCs –CD8^-^CD11b^-^. Additionally, we examine expression of two markers of antigen presentation (CD86 and MHC II) as activation markers on myeloid cell subsets. CD8 T cells were identified as CD45^+^ CD8^+^, CD4 Teff – CD45^+^ CD4^+^ Foxp3^-^, CD4 Treg – CD45^+^ CD4^+^ Foxp3^+^. For T cell differentiation states, naïve T cells were defined as CD62L^+^CD44^-^, central memory T cells as CD62L^+^CD44^+^, effector memory T cells as CD62L^-^CD44^+^. Additionally, we examined expression of several T cell activation markers: Ki67, Granzyme B, and CD25.

For intracellular cytokine staining (IFNγ and TNFα), single cell suspensions of tissues were plated in 96-well round bottom plates containing 200 μL RPMI supplemented with 10% FCS and stimulated with 1 μM PMA (Sigma, St. Louis, MO) and 1 μM Ionomycin (Sigma) for 30 mins at 37°C, then Brefeldin A (1000x dilution, BD Biosciences) and 10 μg/ml Monensin (Sigma) was added and incubated for an additional 5 hr at 37°C. The plates were chilled at 4°C and stained for surface and intracellular markers as described above.

### Statistical analysis

Unless otherwise indicated p values were calculated using a 2-tailed Student’s t test or two-way ANOVA-test. A p value of < 0.05 was considered statistically significant. p values comparing survival curves were calculated using the Log-rank (Mantel-Cox) test. Correlations of immune cell frequencies with tumor weight were done using Pearson’s correlation. All graphs and statistical calculations were done using GraphPad Prism software and Microsoft Excel.

## Results

### RT, VTP and Cryo elicit anti-tumor responses in 4T1 murine breast cancer model

**Figure 1:**
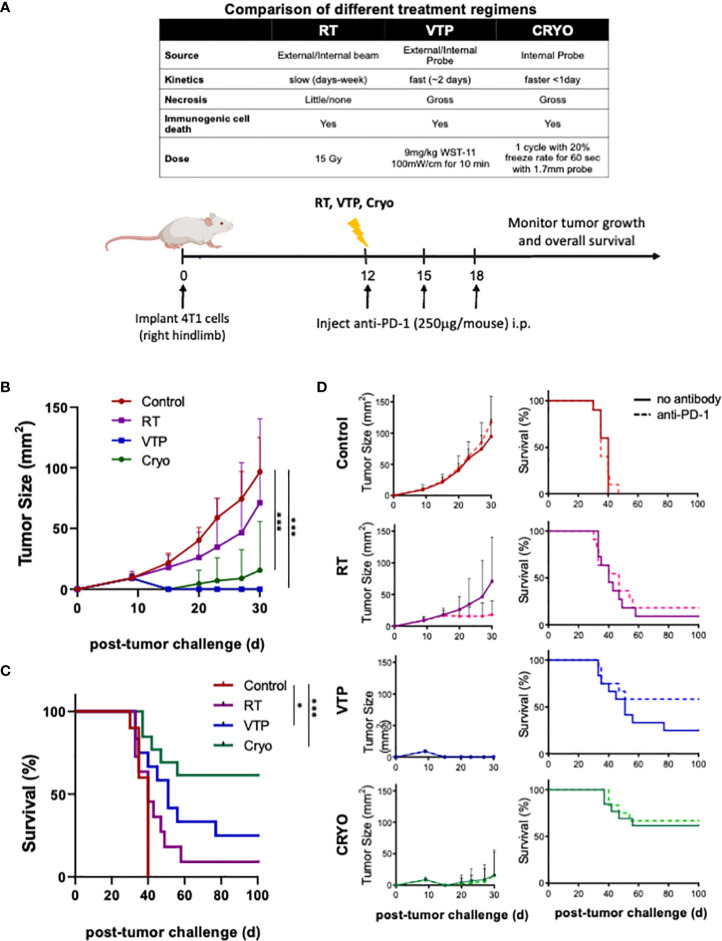
RT, VTP and Cryoablation elicit anti-tumor responses in 4T1 breast cancer. **(A)** Experiment schema of the comparative study with with RT, VTP and Cryo. 2 x 10^5^ 4T1 mouse breast cancer cells were injected subcutaneously in the hind limb of 6-8 week female Balbc mice (n=10 mice per group). 12 days later, mice were treated with RT, VTP or Cryo therapy. For combinations with anti-PD-1, mice were injected intraperitoneally with anti-PD-1 antibody every 3 days for a total of 3 doses. **(B)** Comparison average tumor growth and **(C)** overall survival of 4 cohorts relative to the control. **(D)** Comparison of the average tumor growth +/- SEM and overall survival for individual cohorts with and without anti-PD1 are shown. Statistics were calculated using student T-tests on day 30 for tumor growth and the Logrank method for survival: *p ≤ 0.05, ***p ≤ 0.005.

A summary comparing the kinetics and doses of RT, VTP and Cryo used in this study is listed in the table of **Figure 1A**. To examine the anti-tumor effects of three local ablation therapies, mice implanted with 4T1 breast tumor cells were treated with RT, VTP or Cryo according to the schedule outlined in **Figure 1A**. For the combination treatment with anti-PD-1, mice were also intraperitoneally administered with 3 doses of anti-PD1 antibody at 250 µg/mouse with the first dose given immediately after each treatment. Mice were monitored for tumor growth (**Supplementary Figure S1**; **Figure 1B**) and overall survival (**Figure 1C**). All three therapies delayed tumor growth, with VTP (p<0.0001) and Cryo (p<0.0005) showing statistical significance. VTP and Cryo also improved overall survival (OS) of the animals compared to control group (VTP, p<0.05; Cryo, p<0.0005). RT showed a trend toward improved survival; however, this was not significant (p=0.139). One caveat in comparing these therapies is that treatment with RT and VTP regimens were aimed for suboptimal ablation of the tumors while the dose of Cryo in these studies was optimally ablative due to the nature of the procedure. The addition of anti-PD1 to each of these therapies further delayed tumor growth in a few tumors but had minimal effect on overall survival (**Figure 1D**). The cohort treated with VTP + anti-PD1 trended towards better survival than VTP alone however this difference was not significant (p=0.1663). OS in all three combination cohorts was improved compared to anti-PD1 monotherapy (RT combination vs. anti-PD-1, p<0.088; VTP combination vs. anti-PD-1, p<0.005; Cryo combination vs. anti-PD-1, p<0.0001). Some of the tumors treated with these therapies completely regressed in response to the therapies: two mice in VTP, 7 mice in Cryo, 6 mice in VTP + anti-PD1 combination and 7 mice in Cryo + anti-PD1 combination were tumor free at day 98 post treatment (**Supplementary Figure S1**).

### RT, VTP and Cryo leads to activation of myeloid cells in the tumor, spleen and draining LN

**Figure 2:**
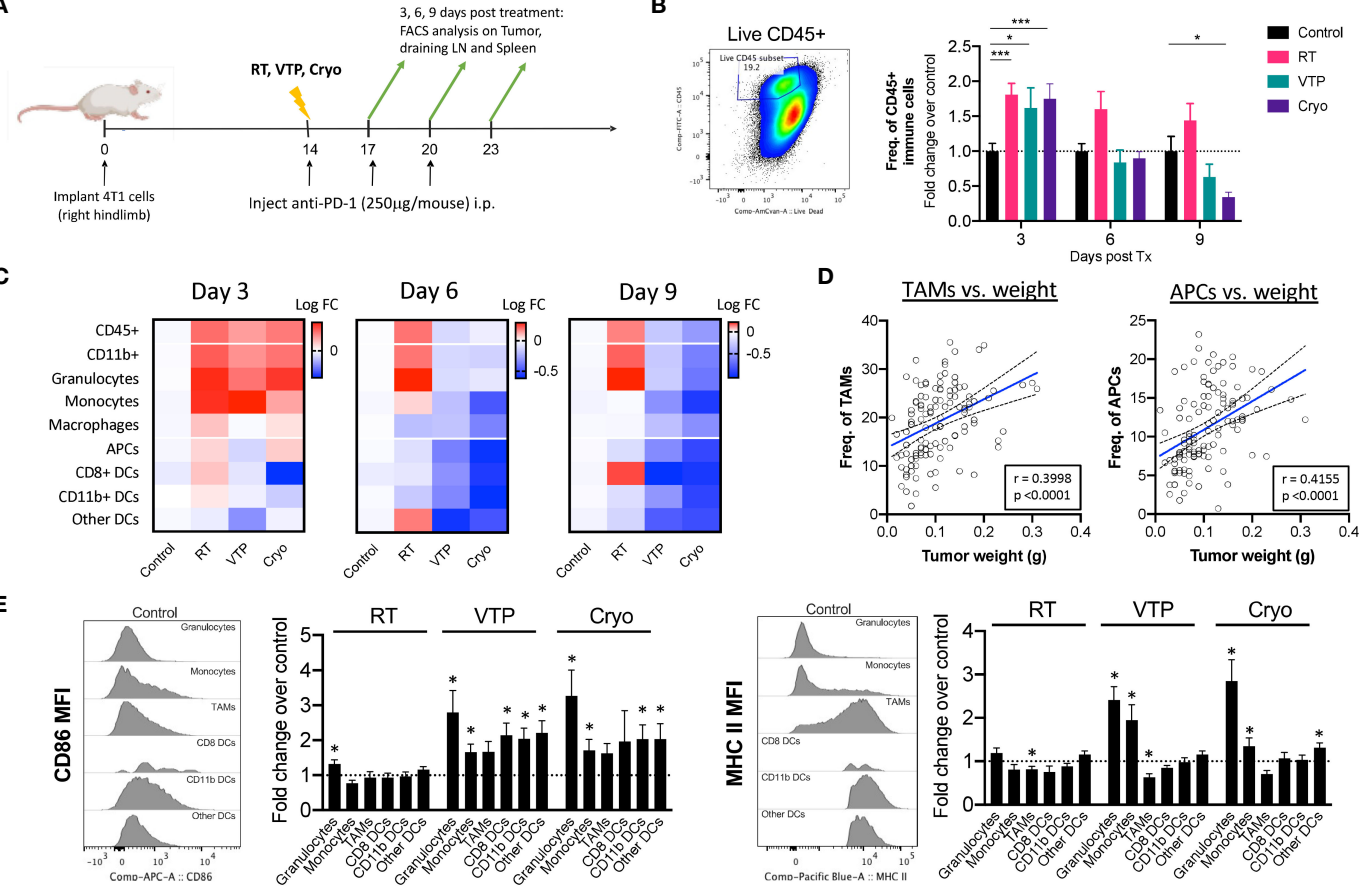
Activation of Myeloid cells in the tumor of animals treated with localized therapies. 2 x 10^5^ 4T1 mouse breast cancer cells were injected subcutaneously in the right hind limb of 6-8 week female Balbc mice (n=5 mice/group). 12 days later when the tumors reached 50-60mm2 in size, mice were treated with 15 Gy radiation (RT), VTP or Cryo therapy according to the doses listed in **Figure 1A** and methods. **(A)** Experiment schema of timeline of tumor challenge, treatment dates and tissue harvest for flow cytometry. **(B)** bivariate plot to show the gating strategy for total immune infiltrates in the tumors (live CD45^+^) and fold change in The frequencies of CD45^+^ cells of each treatment group represented at fold change over the control in the tumor at the indicated time points. **(C)** Heatmaps representing the log10 fold change (Log FC) of the immune cell populations in the tumor. **(D)** Pearson correlations of tumor burden (weight (g)) vs. frequencies of myeloid cells (as a % of CD45^+^ cells) from experiments outlined in **(A)**. **(E)** Histogram plots showing relative expression levels of two activation markers associated with antigen presentation (CD86 and MHC II) on each myeloid cell in the tumor at day 3 post treatment. Bar graphs represent fold change in expression over the control group. Statistics were calculated using a student T-test: *p ≤ 0.05, ***p ≤ 0.005.

To understand the changes in the TME after RT, VTP and Cryo, we performed flow cytometry analysis on tumors, tumor draining lymph nodes (LNs) and spleens isolated from animals treated with RT, VTP and Cryo according to the schedule outlined in **Figure 2A**. Identification of each innate immune cell population was determined using the gating strategy outlined in **Supplementary Figure S2**. We found a significant increase in the frequencies of viable CD45^+^ total immune cells infiltrating the tumors at 3 days after RT, VTP and Cryo (**Figure 2B**). This effect diminished over time in the VTP and Cryo groups quickly however, RT showed different kinetics over time. The increases in immune cell infiltration in the tumor appears to mainly be due to an increase in CD11b^+^ myeloid cells. We found an increase in CD11b^+^ myeloid cells in the tumors at 3 days post all 3 treatments while CD11b^+^ population in spleen and TDLN showed minimal increase (RT) or decrease (VTP & Cryo) (**Figure 2C**; **Supplementary Figure S3**). When CD11b+ cells are sub-gated into granulocytes (CD11b^+^ Ly6G^+^), monocytes (CD11b^+^ Ly6C^+^), and macrophages (CD11b^+^ Ly6G^-^Ly6C^-^), (**Supplementary Figure S2**), we find that the majority of the cells infiltrating the tumors at day 3 are granulocytes and monocytes. These cells are decreased in the tumors treated with VTP and Cryo at 6- and 9-days post treatment. However, they remain higher in the RT treated tumors (**Figure 2C**). In addition, RT was associated with a significant (p < 0.05) increase in CD8^+^ DCs in the tumors at 9 days after treatment (**Figure 2C**). This is consistent with previous reports that local RT activates cross-presenting DCs which elicits CD8^+^ T cell activation (23, 24).

There was an increase although not significant in macrophages (CD11b^+^Ly6G^-^Ly6C^-^F4/80^+^) and antigen presenting cells (APCs: CD11c^+^MHC II^+^) in tumors 3 days after treatment with RT and Cryo, however there is a global decrease in these populations over time (**Figure 2C**). When we examine the correlations of all the innate cells with tumor burden (tumor weight at time of excision), we find that macrophages and APCs have the strongest positive correlation with tumor burden (**Figure 2D**), suggesting that these cells may help promote tumor progression. Of note, all the APC subtypes correlates positively with tumor burden whereas other immune subsets such as monocytes showed no correlation (**Supplementary Figure S4**). While we did observe a decrease in macrophages and APCs over time, the quality of these cells appears to be different after treatment with VTP and Cryo. In fact, most of the innate immune cells showed increased expression of markers associated with antigen presentation after treatment. All the innate cells examined show increased expression of CD86 (B7.2, the co-receptor for CD28 on T cells), however, only neutrophils and monocytes showed increased MHC II expression over the control. This is mainly because macrophages and all the subsets of APCs already express high levels of MHC II in the tumors and this expression does not change after treatment (**Figure 2E**, histogram plots).

Since RT, VTP and Cryo are all localized therapies given directly to the tumors, we sought to determine whether these therapies can elicit a systemic effect on innate immune cells. Consistent with that data from the tumors, we found a reduction of CD11b^+^ population in the spleens (**Supplementary Figure S5A**) and tumor draining LNs (**Supplementary Figure S5B**) treated with VTP and Cryo. The effect was seen as early as 3 days post treatment and persists for up to 9 days post treatment. Similar decreases in APCs were seen in the LNs but not the spleens of treated animals. When we examined expression of activation markers involved in antigen presentation, we found that most of the innate cells in the LN and to a lesser extent in the spleen, showed an increase in expression of CD86 and MHC II. This expression peaks in the LN at 3 days after treatment in the RT groups, and 6 days after treatment with VTP and Cryo. This suggest that the kinetics for RT is different from VTP and Cryo.

### RT, VTP and Cryo leads to activation of T cells in the tumor, spleen and draining LN

In addition to examining cells of the innate immune system, we conducted flow cytometry analysis on the adaptive immune system using markers for CD8^+^ T cells, CD4^+^ effector T cells (Teffs) and CD4^+^ Foxp3^+^ regulatory T cells (Tregs). Tumor bearing animals were treated according to the schedule outlined in **Figure 1A** and tumors, draining LNs and spleens were isolated and processed for flow cytometry according to the timeline in **Figure 2A**. Identification of T cell population and their activation/differentiation states were defined by the gating strategy outlined in **Supplementary Figure S6**. In addition to comparing the effects of RT, VTP and Cryo on the adaptive immune system, we also compared the effects of adding anti-PD-1 to each treatment since anti-PD-1 is known to affect T cell proliferation and activation.

**Figure 3:**
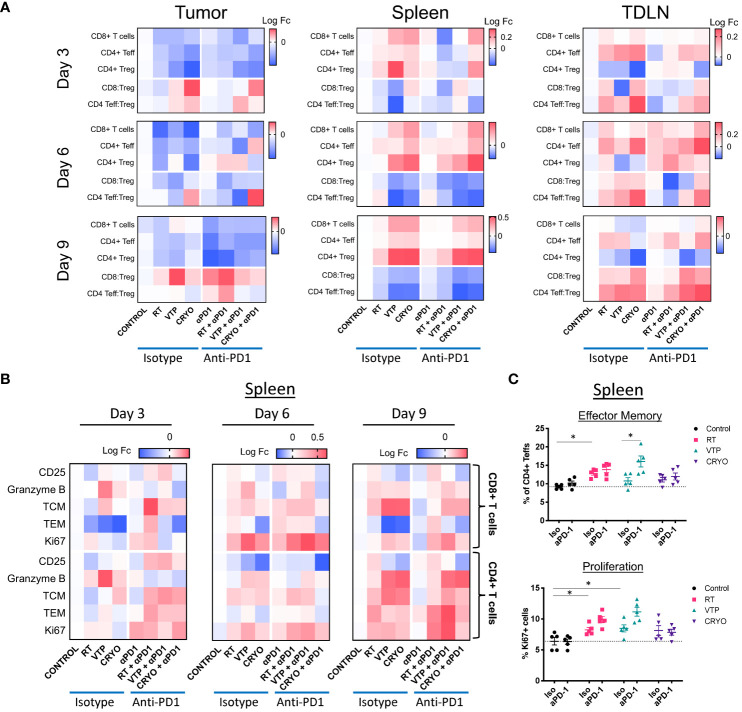
Activation of T cells in the tumor, spleen and draining LN of animals treated with localized therapies. 2 x 10^5^ 4T1 mouse breast cancer cells were injected subcutaneously in the right hind limb of 6-8 week female Balbc mice and treated with RT, VTP or Cryo therapy according to the doses and schedule in **Figure 1A**. 3, 6 and 9 days after the start of treatment, tumor, spleen and draining LNs were harvested and processed for flow cytometry. **(A)** Heatmaps represents the log10 fold change (Log FC) of the CD8^+^ T cells, CD4^+^ Teffs and CD4^+^ Tregs as well as the CD8:Treg and CD4:Treg ratios in the tumor, spleen and LN at 3, 6, and 9 days post treatment. **(B)** Heatmaps represents the log_10_ fold change (Log FC) relative to the control group of T cell activation markers in the spleen with the indicated therapies. **(C)** Quantification of effector memory (Tem) and proliferating (Ki67+) CD4^+^ Teffs (CD4^+^Foxp3^-^) in the spleen 9 days after treatment. *p ≤ 0.05.

We observed a global decrease in all T cell populations in the tumor at all 3 timepoints examined (**Figure 3A**, left and **Supplementary Figure S7**). The relative abundance of the effector T cells (CD8^+^ T cells or CD4^+^ Teffs) with respect to CD4^+^ Tregs can be used to estimate immune responses to immunotherapies. In these experiments even though there is a decrease in these populations, we did observe an increase in the ratio of CD8:Treg and CD4:Treg at day 3 with VTP and Cryo. At day 9, the addition of anti-PD-1 significantly increased the ratio of CD8:Treg in the control, RT and VTP treated groups and there was a trend (not statistically significant) towards an increase in the Cyro group (**Figure 3A**, left and **Supplementary Figure S7**). This effect was especially striking in the RT group where an increase in both CD8:Treg and CD4:Treg was observed with the addition of anti-PD-1. In contrast to the tumors, we found a significant increase in both CD8^+^ T cells and CD4^+^ Teffs in the spleen of treated animals at all time points examined (**Figure 3A**, middle and **Supplementary Figure S8**). However, there was also an increase in CD4^+^ Tregs which led to a decreased CD8:Treg and CD4:Treg ratio. In the tumor draining LNs, there was an early increase in CD8^+^ T cells and CD4^+^ Teffs coupled with an overall decrease in Tregs in all treatment groups. The net effect is an increase in CD8:Treg and CD4:Treg ratios (**Figure 3A**, right and **Supplementary Figure S9**). The addition of anti-PD-1 did not appear to significantly affect the T cell populations in the spleen or LNs.

When we examined the activation status of T cells in the tumor, spleen and LNs at all three time points, we found T cells were activated in the tumor and spleen with slightly different kinetics (**Supplementary Figures S7**, **S8**). No significant changes in T cell activation were observed in the draining LNs (**Supplementary Figure S9**). In some of the conditions, the tumors were at times too small to obtain enough data for statistical power, this was especially the case in the VTP and Cryo treated tumors. However, we did observe some significant changes in the samples which were analyzed. There was an early increase in T cell activation markers mainly on CD8^+^ T cells among tumor samples at 3 days post therapy (**Supplementary Figure S7**). Notably, there was an increase in CD25 (the IL-2Rα chain) in the RT, VTP and Cryo groups. There was also an increase in the cytolytic protein, Granzyme B, expression in CD8^+^ T cells in the RT groups. There was also a significant increase in effector memory (TEM) cells in all the treatment groups. The addition of anti-PD-1 did not significantly enhance the activation state of CD8^+^ T cells or CD4^+^ Teffs in the tumor at any of the time points examined.

There were some significant increases in T cell activation in the spleen at 6- and 9-days post treatment which was most striking in the VTP and Cryo groups (**Supplementary Figure S8**; **Figure 3B**). There was an increase in Granzyme B, and the proliferation marker Ki67 in both CD8^+^ T cells and CD4^+^ Teffs (**Figures 3B, C**; **Supplementary Figure S10A**). There was an increase in both central memory (TCM) and effector memory (TEM) CD4^+^ Teffs at 9 days following all three treatments (**Figures 3B, C**; **Supplementary Figure S10B**). The addition of anti-PD-1 at this time point did not enhance the presence of TCM CD4^+^ Teffs but did enhance the frequencies of TEM CD4^+^ T cells. In addition, anti-PD-1 also enhanced Ki67 on CD4^+^ Teffs in the RT and VTP groups at day 9 (**Figure 3C**). The addition of anti-PD-1 had the strongest effects on T cell activation in the spleen at day 3 post treatment where there was a significant increase in the expression of CD25 in both CD8^+^ T cells and CD4^+^ Teffs in the RT and VTP treated animals (**Figure 3B**; **Supplementary Figure S10C**). There was also an increase in TCM and/or TEM CD8^+^ T cells and CD4^+^ Teffs in all three groups with the strongest changes observed in the RT groups. On day 6, there was an increase in Ki67 in CD8^+^ T cells and CD4^+^ Teffs in animals treated with Cryo and anti-PD-1. In addition, at this time point VTP alone induced a significant increase in Granzyme B expression in both CD8^+^ T cells and CD4^+^ Teffs.

In summary, the changes of T cell activation states in the spleen peaked 6–9 days post treatment with the monotherapies. These observations are highly significant because they show that localized treatments such as RT, VTP and Cryo can have systemic effects on the adaptive immune responses.

### IHC analysis show a decrease in CD11b^+^ myeloid cells and an increase in CD8^+^ T cells in the spleens of animals treated with VTP and Cryo

**Figure 4:**
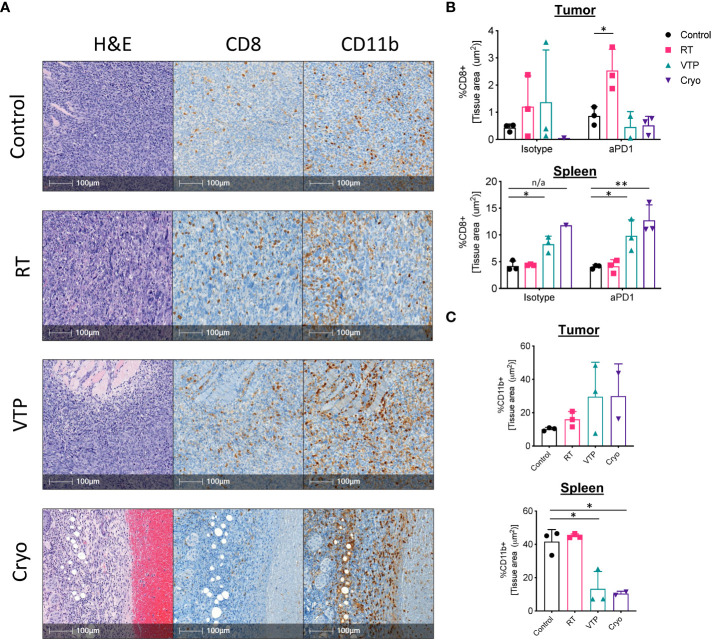
IHC analysis show an increase in CD11b^+^ myeloid cells infiltrating the tumors of animals treated with RT, VTP and Cryoablation. 2 x 10^5^ 4T1 mouse breast cancer cells were injected subcutaneously in the right hind limb of 6-8 week female Balbc mice and treated with RT, VTP or Cryo therapy according to the doses and schedule in **Figure 1A**. 6 days after treatment, tumors and spleens were harvested and processed for immunohistochemistry (IHC). **(A)** Shown are representative images of serial sections for H&E, CD8 and CD11b for each treatment group. Bar = 100µm. **(B, C)** Relative densities of CD8 and CD11b expression in the tumor and spleens. Shown are % of tissue stained positive for each marker calculated and normalized per tissue area using the HALO Image Analysis Software. N=3 mice/group. Statistics were calculated using student’s T-test: *p ≤ 0.05, **p ≤ 0.01. n/a means statistics could not be calculated since the Cryo group had only one analyzable tumor.

While flow cytometry can give us a great deal of information on the breadth and depth of immune cells and their activation state, it cannot assess the spatial distribution of these cells throughout the tissues. To understand the spatial distribution of immune cells in 4T1 breast tumors, we performed immunohistochemistry (IHC) analysis on paraffin embedded sections of spleens and tumors from mice treated with RT, VTP and Cryo. Tumor bearing animals were treated according to the schema outlined in **Figure 1A** and tissues were harvested 6 days after treatment and processed for IHC. Serial paraffin sections were cut and stained with anti-CD8 and anti-CD11b and H&E. From the IHC data, we observed that 4T1 tumors which have not received any treatment are poorly infiltrated with CD8^+^ T cells (**Supplementary Figure S11**; **Figure 4A**). On the contrary, these tumors are more heavily infiltrated by CD11b^+^ myeloid cells, however, many of these cells appear to be confined to the stroma and necrotic tumor areas rather than tumor cell dense areas (**Supplementary Figure S11**). The density in CD11b^+^ myeloid cells in the spleens of 4T1 tumor bearing mice also appear to be high in untreated animals (**Supplementary Figure S12**). These observations are in agreement with the flow cytometry data and previously published data that 4T1 tumors are poorly infiltrated by T cells and highly infiltrated with myeloid cells (2).

Analysis of the IHC data showed that 2/3 RT treated tumors and 1/3 VTP treated tumors showed an increase in CD8 infiltration relative to the control (**Figures 4A, B**). In the anti-PD-1 treated groups, 3/3 RT tumors showed significant increased CD8+ T cell infiltration relative to the control or anti-PD-1 alone (**Figure 4B**). In agreement with the flow data (**Figure 1C**), we also observed a slight increase in CD11b^+^ myeloid cell infiltration into the tumors treated with RT (**Figure 4C**). Because VTP and Cryo destroys the tumor tissue, it is difficult to get large enough healthy tumor areas to effectively quantify immune infiltrates. When the few healthy sections were analyzed, we did not observe any significant changes in CD8+ T cell infiltration into tumors treated with VTP and Cryo (**Figures 4A, B**). Similar to the flow data at early time points, we observed a trend towards an increase in CD11b^+^ cells infiltrating tumors treated with VTP and Cryo (**Figures 4A, C**).

Remarkably, while VTP and Cryo did not appear to induce a T cell response in the tumors, they appear have a strong effect on T cells in the spleen as noted by larger T cell zones in the H&E sections and increased CD8^+^ T cell in the IHC slides (**Supplementary Figure S12**, **Figure 4B**). In addition, VTP and Cryo significantly decrease the CD11b^+^ myeloid population in the spleen (**Supplementary Figure S12**, **Figure 4C**). These data support the conclusion that that localized treatments such as VTP and Cryo can have systemic effects on the immune system.

### RT, VTP and Cryo led to increased cytokine production by CD4+ and CD8+ T cells in the spleen

**Figure 5:**
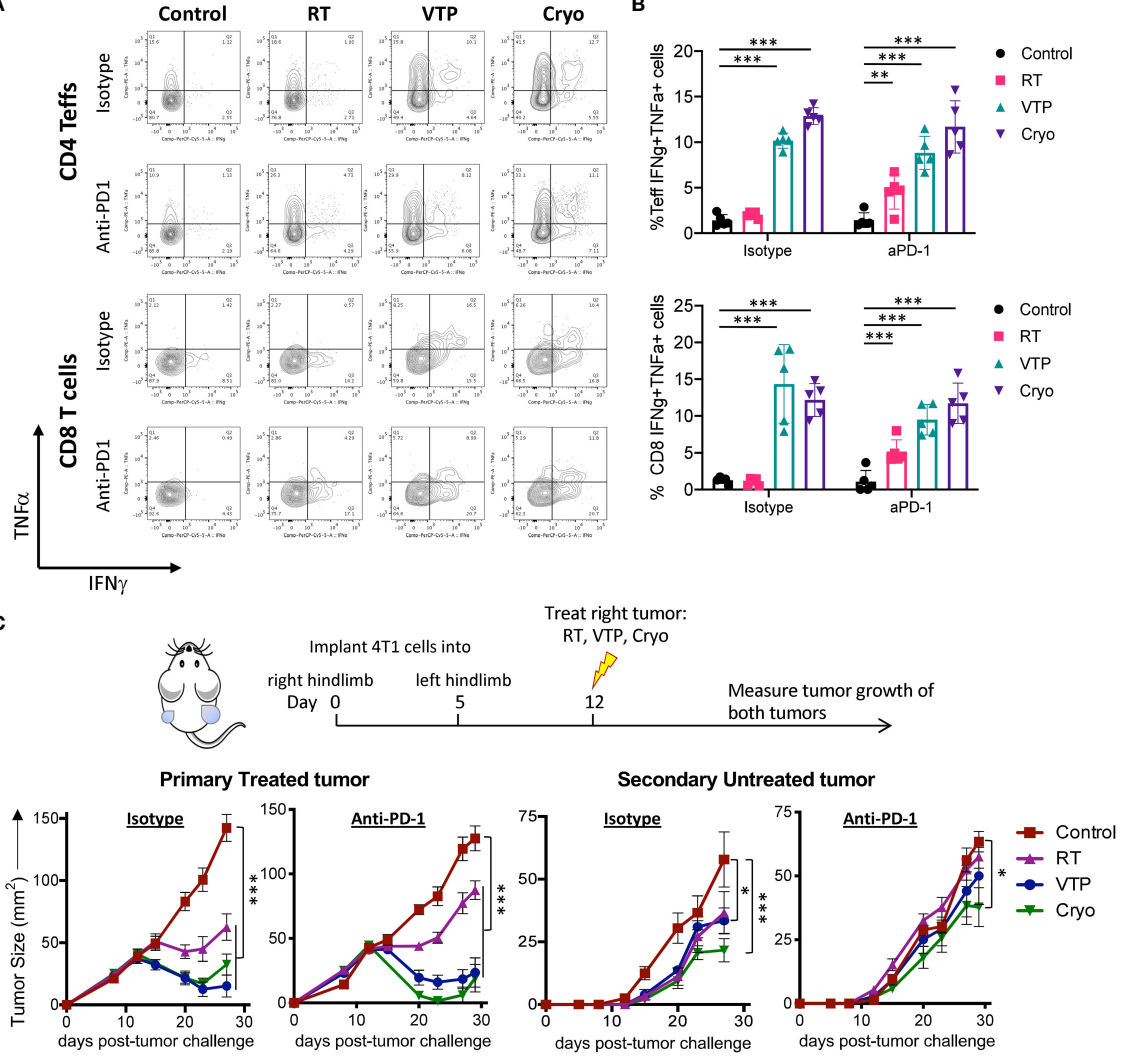
RT, VTP and Cryoablation incudes systemic T cell activation and promotes regression of secondary tumors. **(A, B)** 2 x 10^5^ 4T1 cells were injected subcutaneously in the right hind limb of 6-8 week female Balbc mice (5 mice/group) and treated with RT, VTP or Cryo therapy according to the doses and schedule in **Figure 1A**. 6 days after treatment, tumors, LNs and spleens were harvested and single cells suspension were stimulated with PMA/Ionomycin in the presence of Golgi inhibitors for 6hr then processed for intracellular cytokine staining (ICS). **(A)** Representative plots of IFNγ vs. TNFα by CD4^+^ Teffs and CD8^+^ T cells from the spleen. **(B)** Frequencies of IFNγ^+^TNFα^+^ CD4^+^ Teffs and CD8^+^ T cells in the spleen. **(C)** 10^5^ 4T1 cells were injected subcutaneously in the right hind limb on day 0 and left flank on day 5 of female Balbc mice (10 mice/group). Twelve days after the initial tumor injection, the right hind limb tumors were treated with 15 Gy RT, VTP or Cryoablation as outlined in the treatment schedule. Tumor size was measured using calipers, shown are the average tumor size (mm^2^) +/- SEM in both primary treated and secondary non-treated tumors. Statistics were calculated using a student T-test. For tumor growth curves, statistics were calculated on day 29 post tumor inoculation. *p ≤ 0.05, **p ≤ 0.01, ***p ≤ 0.005.

Our findings that T cell activation markers are increased on CD4^+^ and CD8^+^ T cells in the spleens of animals treated with localized therapies such as RT, VTP and Cryo suggests a systemic effect of these therapies on the adaptive immune system. To determine whether these phenotypic increases translate into a function change by T cells, we stimulated single cell suspensions isolated from the spleens of treated animals with PMA and Ionomycin in the presence of Golgi inhibitors (Brefeldin A and monensin) to examine the T cells’ ability to produce pro-inflammatory cytokines such as interferon-gamma (IFNγ) and tumor necrosis factor-alpha (TNFα). We also examined if these T cells can produce both IFNγ and TNFα simultaneously. These polyfunctional T cells have been shown to be the most functional effector cells (25). We found that there was an increase in both IFNγ^+^ and TNFα^+^ CD4^+^ and CD8^+^ T cells as well as IFNγ^+^TNFα^+^ double positive polyfunctional T cells in the spleen in response to RT, VTP and Cryo. The most robust increases seen in the VTP and Cryo groups (**Figures 5A, B**). The addition of anti-PD-1 to these treatments significantly increased the cytokine production of T cells in the RT groups but did not further enhance the effects of VTP and Cryo (**Figure 5B**). These data suggest that all three therapies elicited systemic effector T cell responses.

### RT, VTP and Cryo delayed growth of secondary tumors in a bilateral tumor model

The finding that RT, VTP and Cryo can induce a systemic immune response suggest that these therapies may be capable of inducing an abscopal effect by controlling the growth of distant tumors or metastases. To assess whether these treatments can elicit regression of distant tumors, we used a bilateral tumor model where the tumor implanted at 12 days prior to treatment is treated and the second implanted at 7 days prior to treatment is left untreated (**Figure 5C**). We monitored growth of the treated and untreated tumors over time. We found that all three modalities (RT, VTP, Cryo) delayed the growth of both the treated and untreated tumors (**Figure 5C**) with Cryo having the greatest effect on the secondary tumors. However, the addition of anti-PD1 to the treatment did not affect the secondary tumor growth. This data is consistent with the fact that anti-PD1 has modest effects on enhancing activation of T cells when combined with these treatment regimens (**Figures 3**, **4A, B**; **Supplementary Figures S7**–**S9**).

## Discussion

Here we show that while RT, VTP and Cryo have been all shown to induce immunogenic cell death, they have differential effects on the adaptive and innate immune system. In general, VTP and Cryo have similar effects while RT appears to be different. One explanation for this may be the differences between the kinetics in which these treatments work. RT induces cell death through DNA damage and subsequent apoptosis or mitotic catastrophe, but these effects may take days to weeks to manifest, and the effects continue for weeks to months after therapy ends. Necrosis can be seen within tumors as well post RT, but at a relatively low frequency (26). There are parallel effects to the radiosensitive capillaries lined with endothelial cells, which dysregulates the balance of pro- and anti-inflammatory factors. Radiation-induced vascular damage increases the expression of ICAM-1 and E-selectin, which are adhesion molecules that mediate inflammatory reactions and promote recruitment of macrophages (27). The kinetics of VTP and Cryo are much more rapid and changes to the tumor (e.g., changes to the endothelium and tissue destruction) can be observed within hours of treatment. For VTP treatment, production of short-lived super oxide and hydroxyl radicals initiate rapid destruction of the targeted vasculature followed by coagulative necrosis of the tumors (12, 28). Recent studies using non-invasive optical imaging such as raster-scanning optoacoustic mesoscopy (RSOM) (28) and Multispectral Optoacoustic Tomography (MSOT) (29) have revealed that vascular destruction by VTP occurs from minutes up to an hour in subcutaneous murine tumor models (CT26 colon and RENCA renal tumor) followed by eschar, edema and tumor necrosis at 48 hours after VTP. By 5 days, there was no visible tumor detected at an optimal treatment condition. With Cryo, cellular damage and death is immediate through rapid freezing and thawing, with subsequent tissue necrosis. In the periphery of the treatment zone, further effect is seen with apoptotic cell death in tissue not exposed to immediately lethal temperatures. There is also evidence of a vascular effect, as ice crystal formation within blood vessels damages the endothelium, which comes into contact with platelets in the reperfusion period, leading to thrombus formation and delayed ischemia, as well as inflammatory cytokine release that increases vascular permeability (30). Additionally, the tumor growth delay induced by VTP and Cryo appears to be more rapid as seen in **Figures 1B, D**.

One of the most significant observations from this study is the decrease in myeloid cells after treatment with VTP and Cryo. The populations that we phenotypically defined as granulocytes and monocytes based on their expression of CD11b, Ly6G and Ly6C are also known as granulocytic MDSCs (G-MDSCs) and monocytic MDSCs (31). These populations when isolated from 4T1 tumors have been shown to be immunosuppressive (2, 3). VTP and Cryo significantly decrease the presence of MDSCs in 4T1 tumors and in the spleens of tumor bearing animals. This also correlated with an increase in CD4^+^ and CD8^+^ T cell responses such as increase in expression of T activation markers and cytokine production (**Figures 5A, B**). However, we observed mixed responses of Tregs with these different modalities. There was a decrease in Tregs in the tumors and draining LNs after treatment with all three therapies, which was linked to an increase in the effector:Treg ratios in both tissues with VTP and Cryo and in the LNs only with RT. However, there was an overall increase in Tregs in the spleens of treated animals in all three treatment modalities. Tregs have been shown to play a role in the recruitment of immunosuppressive myeloid cells in the tumors (32), therefore it is not surprising that both Tregs and MDSCs are decreased in the tumors after treatment with VTP and Cryo.

TNBC is also an attractive model for novel therapies such as VTP and Cryo. Unlike other subtypes of breast cancer, it has limited therapeutic options. We selected 4T1 breast cancer as the preclinical model for this study because it is one of the most well characterized models for TNBC. And like TNBC in patients, 4T1 tumors have been shown to be poorly responsive to ICB. While there are very few alternatives for preclinical models for TNBC other than 4T1, they are less characterized. Therefore, one potential limitation of this study is that we have only one model of TNBC. 4T1 tumors have poor T cell infiltration and high MDSC infiltration (2). Mechanisms that target immunosuppressive myeloid cells have been shown to re-sensitize 4T1 tumors to ICB (2). In this study, the decrease in myeloid cells after VTP and Cryo, while significant, did not sensitize 4T1 tumors to ICB with anti-PD-1 in concert with these treatments using this regimen. The antitumor efficacy of anti-PD-1 in combination with RT, VTP and Cryo was modest and for the most part not significant. Since 4T1 tumors are highly infiltrated by MDSC and immunosuppressive macrophages (2), we hypothesize that there are still significant amounts of the immunosuppressive cells remaining in the tumors to restrict the T cell responses induced by anti-PD1. On day 9 post therapy, there was approximately a 27% decrease with VTP and 66% decrease with Cryo of the total myeloid (CD11b^+^) cells in the tumors on day 9. One way to potentially circumvent this problem is to combine these therapies with myeloid depleting or repolarizing regimens such as PI3K gamma inhibition (2) or CSF-1R blockade (33, 34).

We also found evidence of the abscopal effect, which is a phenomenon where localized treatment of a tumor causes regression of distant untreated metastatic tumors. This phenomenon has been extensively described in patients undergoing ionizing RT, and it is known that the immune system plays a major role. However, the abscopal effect is not limited to RT. In fact, any localized treatment can induce a systemic immune response that can regress secondary tumors. An important finding of this study is that tumor directed therapies such as RT, VTP and Cryo can control the growth of distal untreated tumors in a bilateral tumor model. The delay in growth of the distal untreated tumors by these therapies suggests that a systemic immune response to tumor antigen was induced by these modalities. Data from the spleen and LNs of treated animal also show activation of T cells in response to RT, VTP and Cryo. These include increase in T cell activation markers, memory markers and cytokine production. Similar to what was observed in the single tumor models, the addition of anti-PD-1 did not have any significant effects of delaying growth of the secondary untreated tumors.

While we have demonstrated the variations in the immunomodulatory effects of these three local tumor ablation options, there are important differences in the clinical applicability of each treatment method, with implications on future therapeutic designs. RT can theoretically be used to target any tumor location within the body, but there are collateral damage risks to surrounding organs (26). Moreover, an important limitation to the usage of RT is the cumulative body dose limit, and so RT cannot be delivered to the same patient in indefinite amounts (35). In contrast, VTP, which has expanding indications with clinical trials investigating its utility in the treatment of prostate (36–38) (NCT03315754), esophageal (NCT03133650), and upper tract urothelial cancers (NCT04620239), has more tumor-selective properties by exploiting the increase in vasculature, and thus uptake of photosensitizer, within tumors. The treatment effect is not by direct cell damage, as seen with RT and Cryo, but by the creation of ROS that collapse the tumor vasculature. Thus, the risk of collateral damage is also less, which confers a particular advantage to the treatment of endoluminal tumors such as in the upper urothelial tract, where the consequences of collateral damage (ureteral stricture or obliteration and renal obstruction) are very high (39). Cryo has been applied to cervical, eye, kidney, liver, lung, and prostate cancers, and it is also an option for reducing bone pain and in local tumor control of bone and soft tissue oligometastasis (40–42). However, treatment is mostly done percutaneously or laparoscopically, which is a major limitation to Cryo since target tumors need to be reachable by the cryoprobe (30). In patients with underlying emphysema, cryoablation may increase the risk of pneumothorax and bleeding, which could be detrimental in patients with poor pulmonary reserve. Compared with heat-based thermal ablation therapies, VTP (non-thermal) and cryoablation (freezing) preserve the collagenous tissue and are safer options near large vasculature (43). Both therapies can be performed multiple times (VTP: UTUC Phase 1) and if necessary, can be followed by salvage therapies. Lastly, RT is typically fractionated into separate doses due to the slower proliferation of normal versus tumor tissues, which confers a survival advantage to normal tissue by better repairing sublethal radiation damage. However, this requires multiple doses of RT, typically over several weeks. On the other hand, VTP and Cryo can be performed in one outpatient sitting, albeit under sedation or anesthesia.

In summary, any means of local tumor destruction that was studied here can provide an *in-situ* vaccination effect that can generate a systemic adaptive immune response. The specificity and timing of each intervention should be taken into consideration when designing therapeutic interventions and combination therapies.

## Data availability statement

The original contributions presented in the study are included in the article/**Supplementary Material**. Further inquiries can be directed to the corresponding authors.

## Ethics statement

The animal study was approved by IACUC of Memorial Sloan Kettering Cancer Center. The study was conducted in accordance with the local legislation and institutional requirements.

## Author contributions

SB: Writing – review & editing, Writing – original draft, Visualization, Methodology, Investigation, Formal analysis, Data curation, Conceptualization. KK: Writing – review & editing, Writing – original draft, Visualization, Validation, Methodology, Investigation, Formal analysis, Data curation, Conceptualization. WY: Writing – review & editing. SR: Writing – review & editing, Methodology, Investigation, Data curation. SJ: Writing – review & editing, Validation, Methodology, Investigation, Data curation. LC: Writing – review & editing, Methodology, Investigation, Data curation. AH: Methodology, Investigation, Formal analysis, Data curation, Writing – review & editing. JT: Methodology, Investigation, Data curation, Writing – review & editing. DP: Data curation, Writing – review & editing. ASo: Methodology, Investigation, Data curation, Writing – review & editing. BG: Methodology, Investigation, Data curation, Writing – review & editing. ASc: Methodology, Conceptualization, Writing – review & editing. JW: Supervision, Resources, Funding acquisition, Conceptualization, Writing – review & editing. JE: Resources, Methodology, Investigation, Writing – review & editing. TM: Methodology, Investigation, Funding acquisition, Formal analysis, Conceptualization, Writing – review & editing, Writing – original draft, Supervision, Resources. JC: Writing – review & editing, Writing – original draft, Supervision, Resources, Methodology, Investigation, Funding acquisition, Formal analysis, Conceptualization.
