# Supplementary material for: Comparative study of immune response to local tumor destruction modalities in a murine breast cancer model
Source: Front Oncol. 2024 Jun 18;14:1405486. doi: 10.3389/fonc.2024.1405486 (PMC11217310; doi:10.3389/fonc.2024.1405486)
Supplement: Supplementary file 1 [file Presentation_1.pdf]

Comparative study of immune response to local tumor destruction modalities in a murine breast cancer model

Supplementary Figures

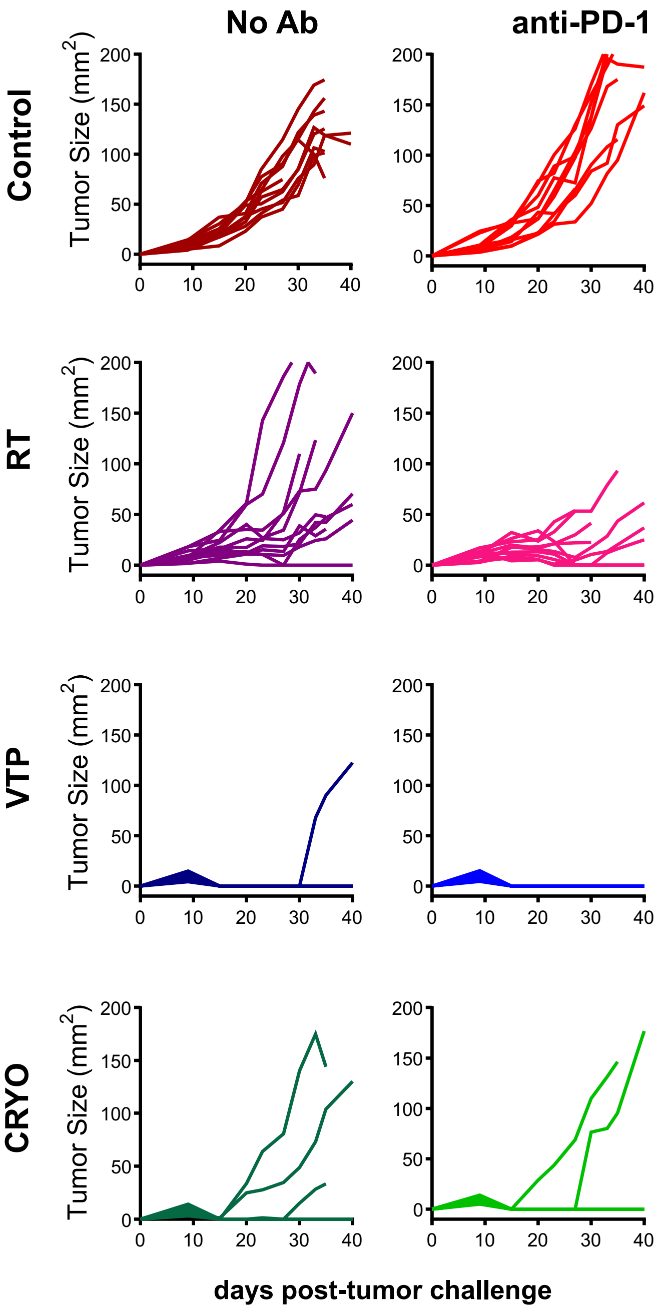

Tumor free mice at day 98		
	No Ab	Anti-PD1
Control	0	0
RT	1	1
VTP	2	6
Cryo	7	7

Fig. S1: RT, VTP and Cryoablation enhances anti-tumor responses in 4T1 breast cancer. Balbc mice were implanted with 4T1 tumors and treated according to the schema in Figure 1A and as described in methods. Shown are individual tumor growth curves (n=10 mice per group) for 8 cohorts are shown. Table: tumor free numbers were assessed at day 98 post implantation.

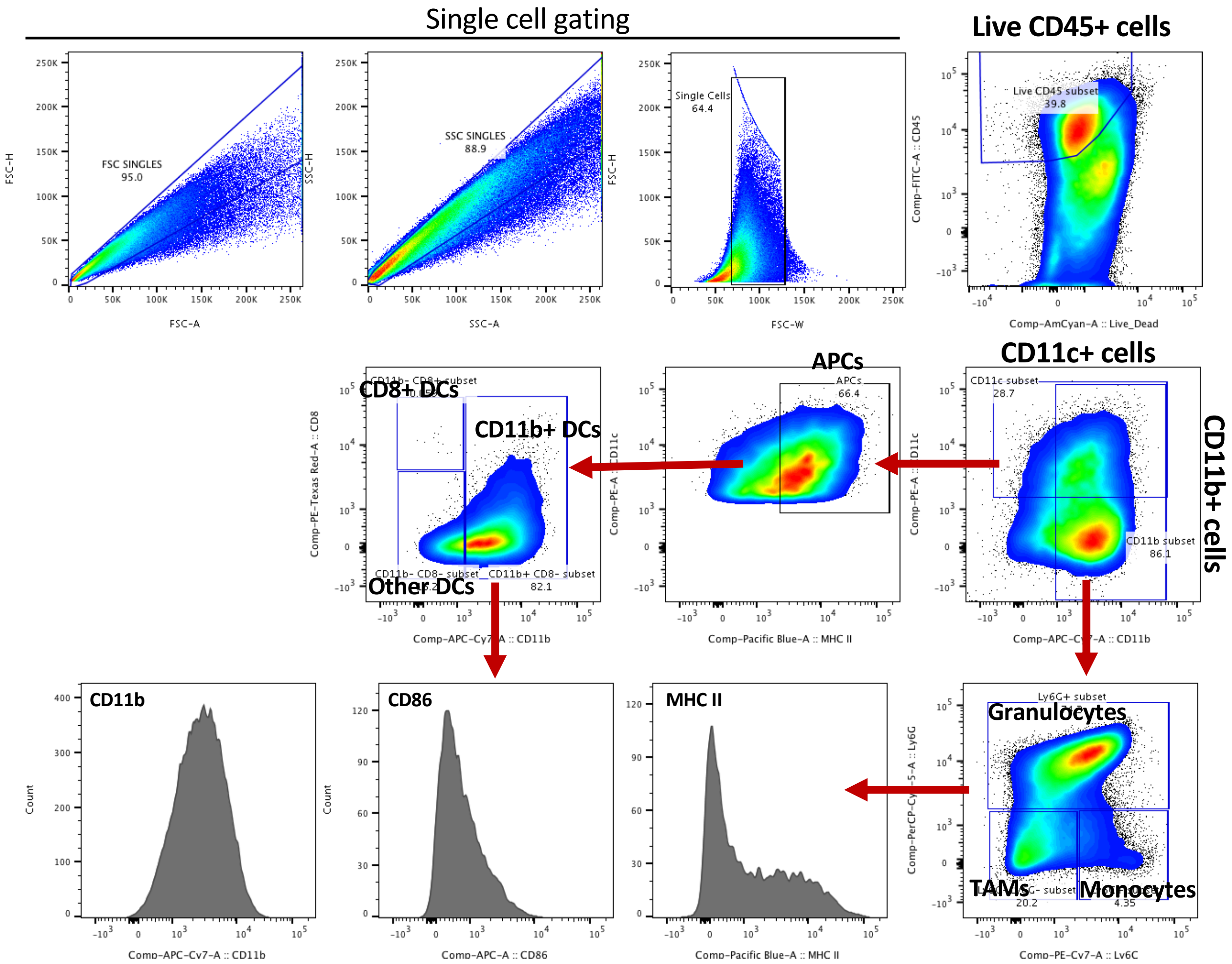

Fig. S2: Gating strategy to identify each innate immune cell population in tissues. Shown is the gating strategy of a representative tumor sample to identify each myeloid cell population and their activation markers. First is to gate on single cells only using FSC-H vs FSC-A, then SSC-H vs SSC-A, then FSC-H vs FSC-W. Next is to gate on the live CD45+ cells for total immune infiltrates. Then the CD45+ cells are sub-gated into CD11b+ and CD11c+ cells. CD11b+ cells are then sub-gated into Ly6G+ (granulocytes), Ly6C+ (monocytes), and Ly6G- Ly6C- cells (mostly macrophages and DCs, here referred to as TAMs). The CD11c+ cells are subdivided into CD8+ DCs, CD11b+ DCs and other DCs. Then the activation status of each cell population is examined by expression of CD11b, CD86 and MHC class II on their cell surface. CD86 and MHC II are used as surrogate for increased antigen presentation while CD11b is used as an activation marker.

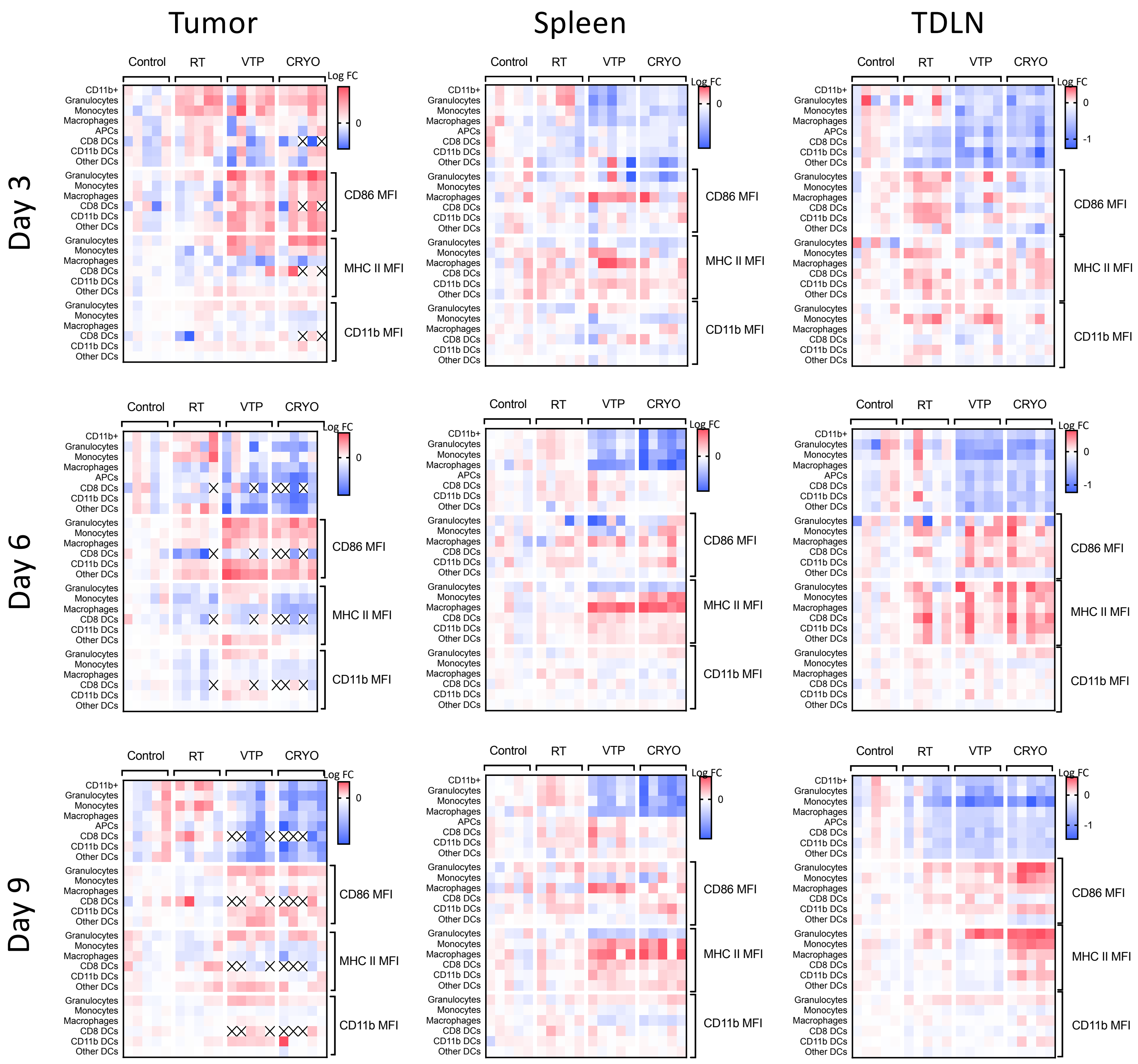

Fig. S3: Summary of myeloid cell kinetics and activation status in the spleen, draining lymph node (TDLN) and tumor. Shown are heatmaps of the $\log_{10}$ fold change (Log FC) of the cell populations and their activation markers for each treatment group normalized to the control. The gating strategy to identify each cell population is shown in Figure S2.

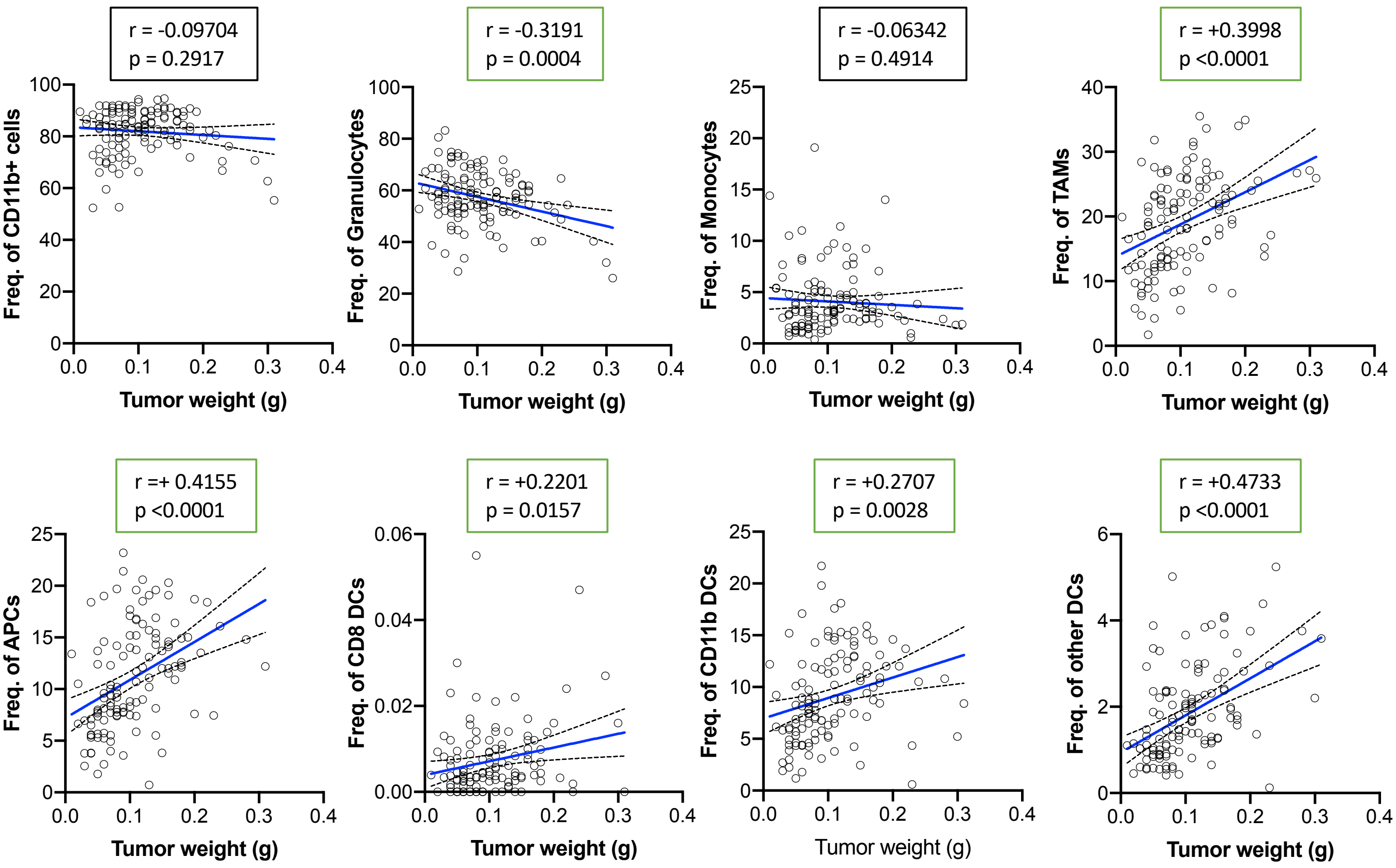

Fig. S4: TAMs and APCs correlated positively with tumor burden. Plots of frequencies of myeloid cells (as a % of CD45+ cells) vs. tumor burden (weight (g)) from experiments outlined in Figure 1A. Each plot represents pooled data from all treatment groups and all time points examined (n=120). Pearson correlation was used to determine the r and p values. The green boxes indicate the correlations that were statistically significant.

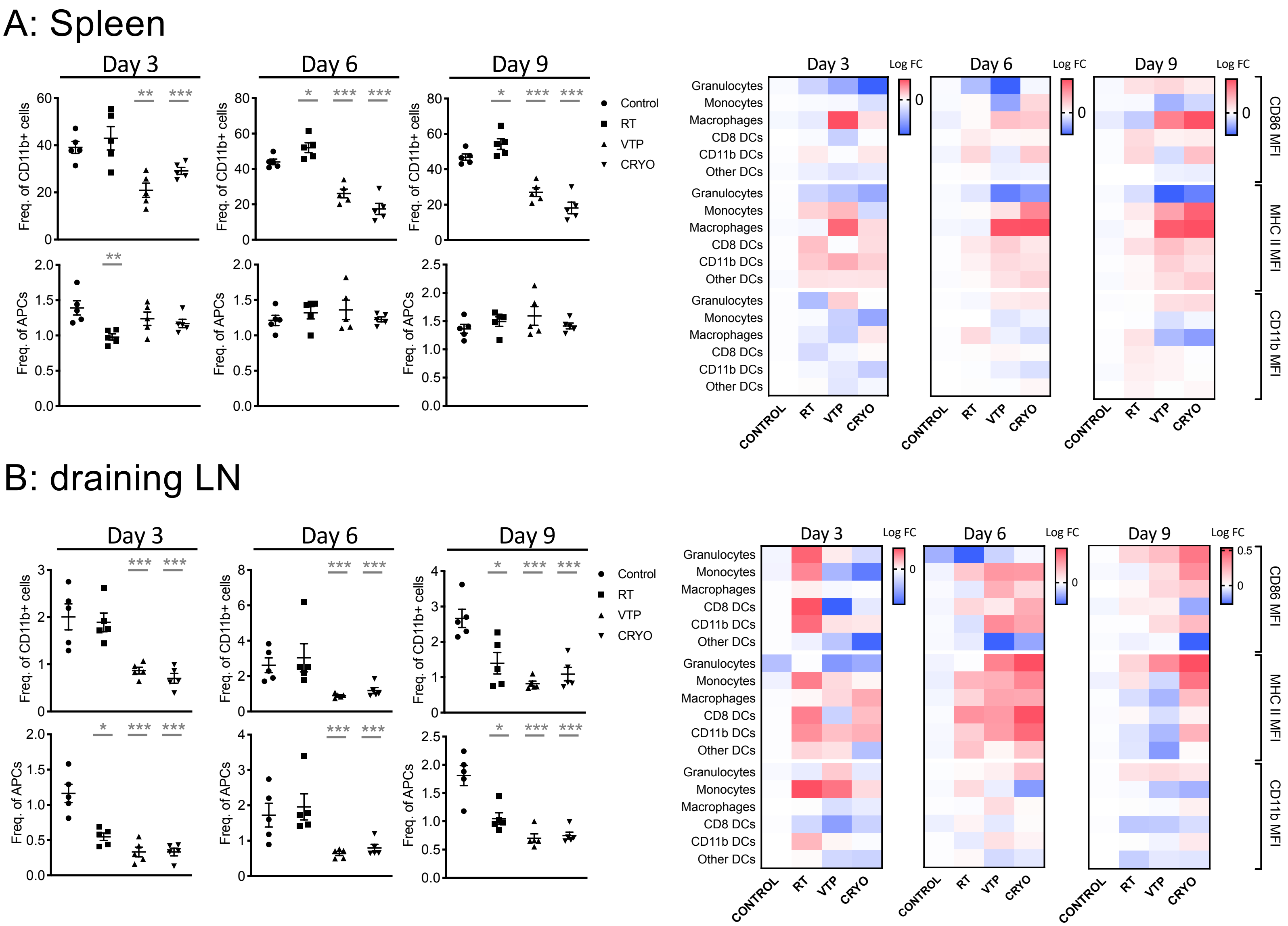

Figure S5: Activation of Myeloid cells in the spleen and draining LN of animals treated with localized therapies. 10^5 4T1 mouse breast cancer cells were injected subcutaneously in the right hind limb of 6-8 week female Balbc mice (n=5 mice per group). Ten days later when the tumors reached 50-60mm² in size, mice were treated with 15 Gy radiation (RT), VTP or Cryo therapy according to the doses listed in Fig 1A and methods. (A,B) *Left:* The frequencies +/- SEM of CD11b+ myeloid cells and CD11c+MHC II+ APCs (as a % of CD45+ cells) in the spleen (A) and draining LN (B) from each treatment group at the indicated time points. *Right:* Heatmaps representing the fold change (Log FC) of CD86, MHC II and CD11b expression based on mean fluorescence intensity (MFI) on immune cells in the spleen (A) and draining LN (B). *p ≤ 0.05, **p ≤ 0.01, ***p ≤ 0.005.

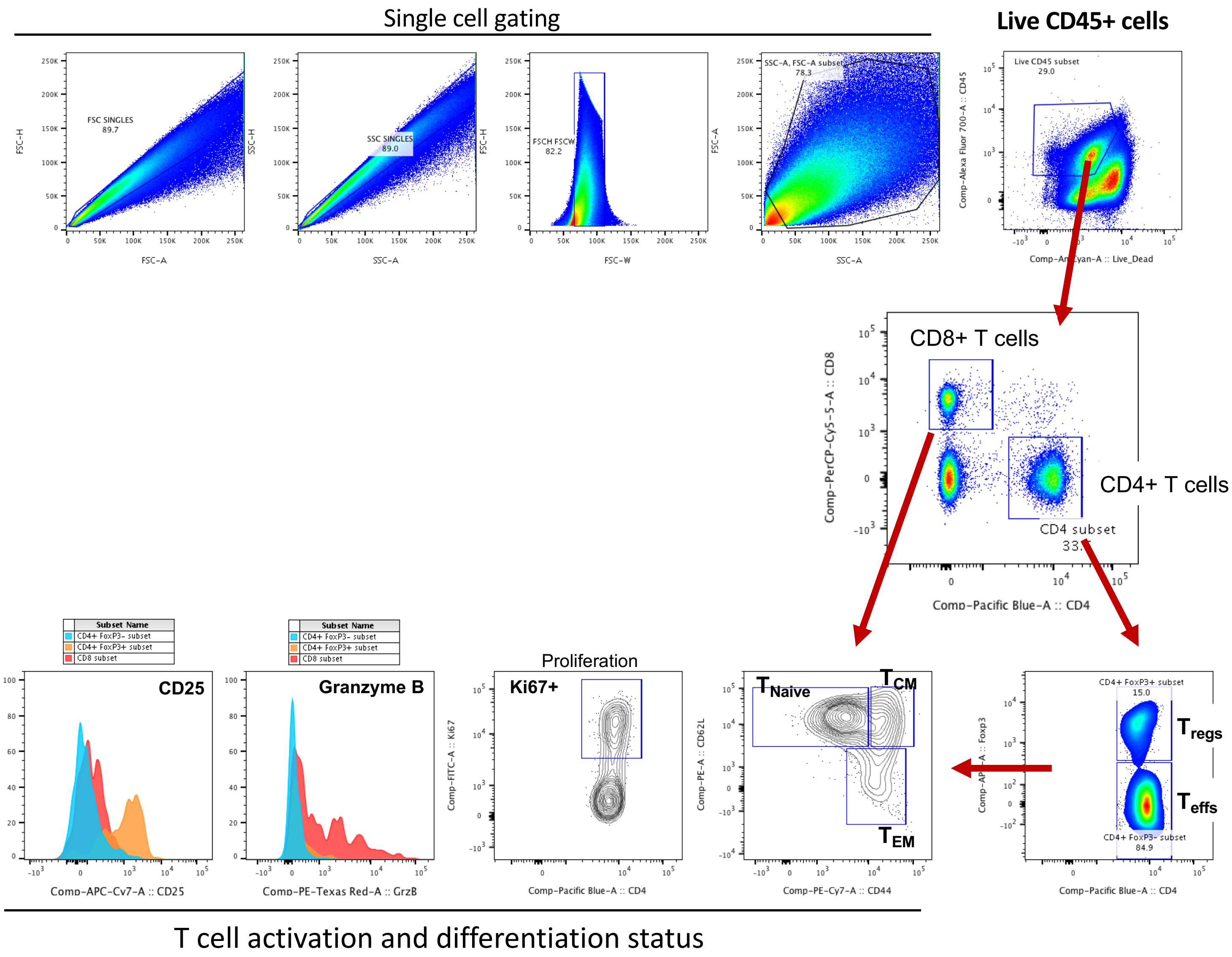

Fig. S6: Gating strategy to identify each T cell population and their activation/differentiation status. Shown is the gating strategy of a representative tumor sample to identify each T cell population and their activation markers. First is to gate on single cells only using FSC-H vs FSC-A, then SSC-H vs SSC-A, then FSC-H vs FSC-W. Next is to gate on the live CD45+ cells for total immune infiltrates. Then the CD45+ cells are sub-gated into CD4+ and CD8+ T cells. The CD4+ cells are further divided into Tregs and Teffs based on Foxp3 expression. All three T cell populations (CD8+ T cells, CD4+ Teffs and CD4+ Tregs) are then examined for their differentiation status by CD62L and CD44 expression. Naïve T cells are CD62L+CD44-, central memory T cells (T_{CM}) are CD62L+CD44+ and effector memory T cells (T_{EM}) are CD62L-CD44+. The T cell populations are further examined for the proliferation (Ki67) and activation status (Granzyme B, CD25).

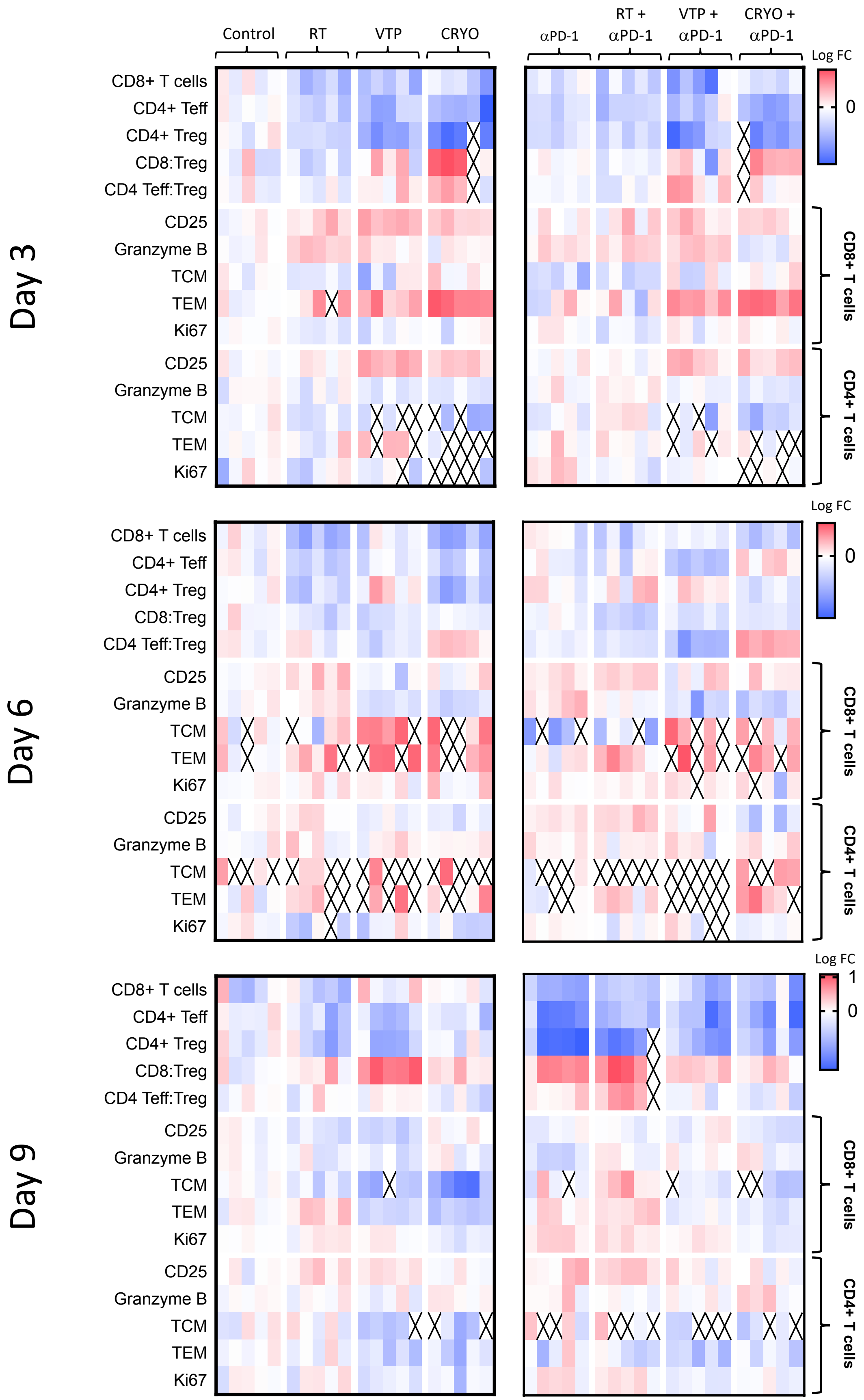

Figure S7. Heatmap summary of T cell frequencies and their activation status in the tumor. Heatmaps from the experiments outline in Figure 1 schema, shown are the $\log_{10}$ fold change (Log FC) of T cell populations and their activation markers for each treatment group normalized to the control. The gating strategy to identify each cell population is shown in Figure S6.

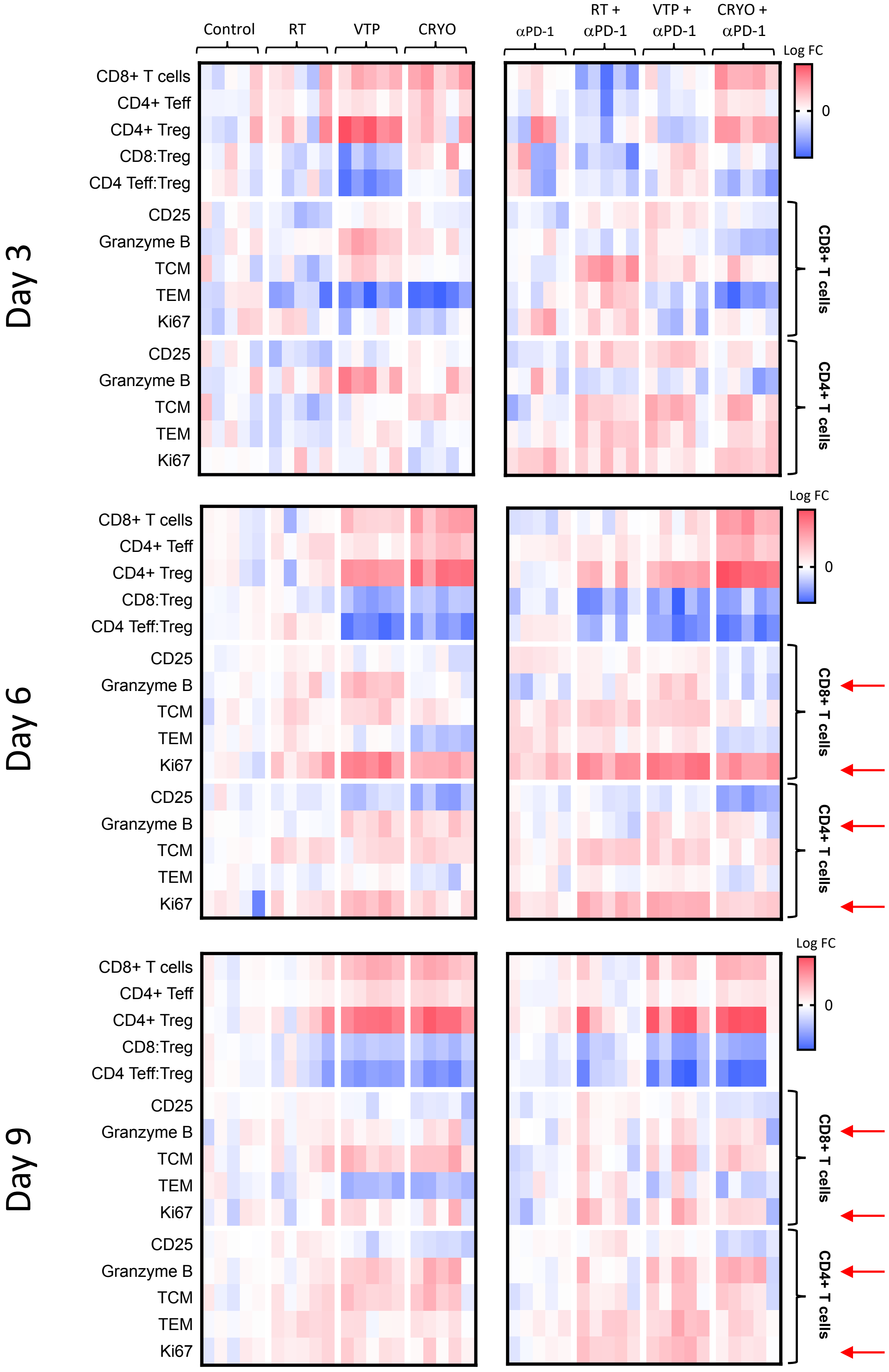

Figure S8. Heatmap summary of T cell frequencies and their activation status in the spleen. Heatmaps from the experiments outline in Figure 1 schema, shown are the $\log_{10}$ fold change (Log FC) of T cell populations and their activation markers for each treatment group normalized to the control. The gating strategy to identify each cell population is shown in Figure S6. Red arrows highlight significant changes observed in Granzyme B and Ki67.

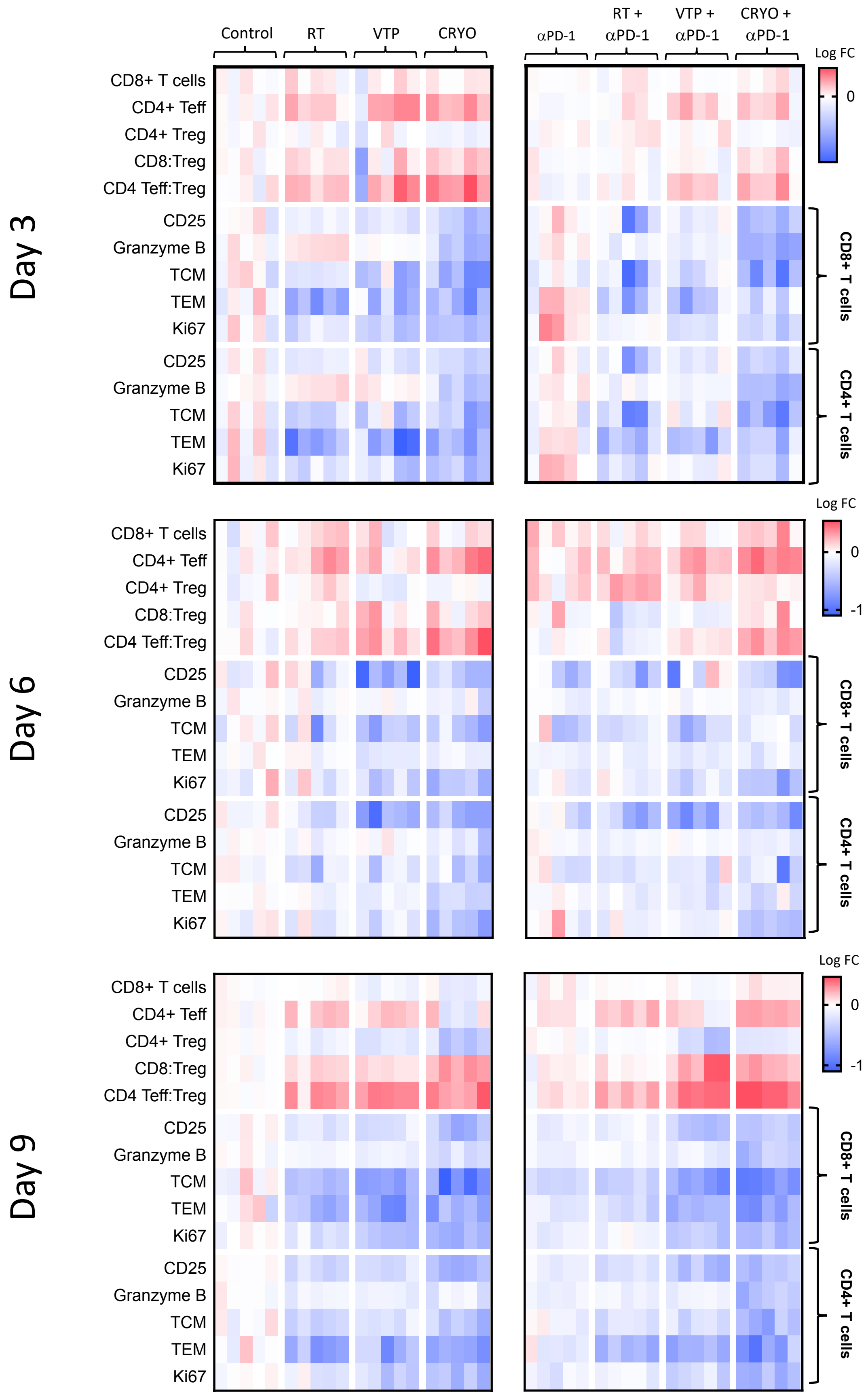

Figure S9. Heatmap summary of T cell frequencies and their activation status in the darning LN. Heatmaps from the experiments outline in Figure 1 schema, shown are the $\log_{10}$ fold change (Log FC) of T cell populations and their activation markers for each treatment group normalized to the control. The gating strategy to identify each cell population is shown in Figure S6.

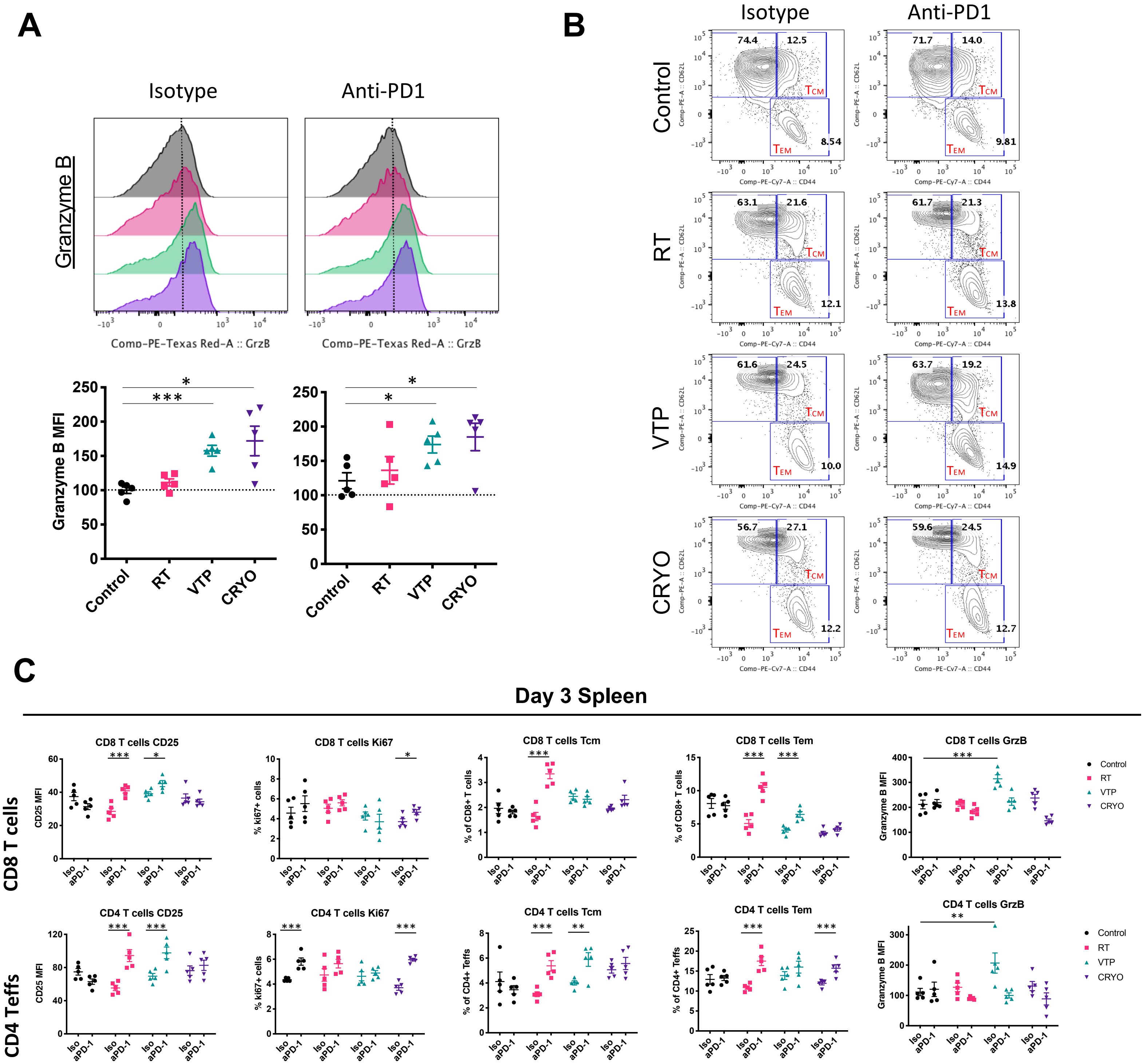

Figure S10: Activation of T cells in the spleen of animals treated with localized therapies. 10^5 4T1 mouse breast cancer cells were injected subcutaneously in the right hind limb of 6-8 week female Balbc mice and treated with RT, VTP or Cryo therapy. 3, 6 and 9 days after the start of treatment, tumor, spleen and draining LNs were harvested and processed for flow cytometry. (A) Representative histogram plots and quantification of Granzyme B expression on CD8 T cells in the spleen 9 days post treatment. (B) Bivariate plots of CD44 vs. CD62L to display CD4+ Teff central memory (Tcm, CD44+CD62L+) and effector memory (Tem, CD44+CD62L-) cells in the spleen 9 days post treatment. (C) Plots represent MFI (CD25, Granzyme B) and frequencies of Ki67+, Tcm, and Tem of CD8+ T cells (Top) and CD4+ Teffs (bottom) in the spleen 3 days post treatment with the indicated therapies +/- anti-PD-1. Statistics were calculated using a student T-test: *p ≤ 0.05, **p ≤ 0.01, ***p ≤ 0.005.

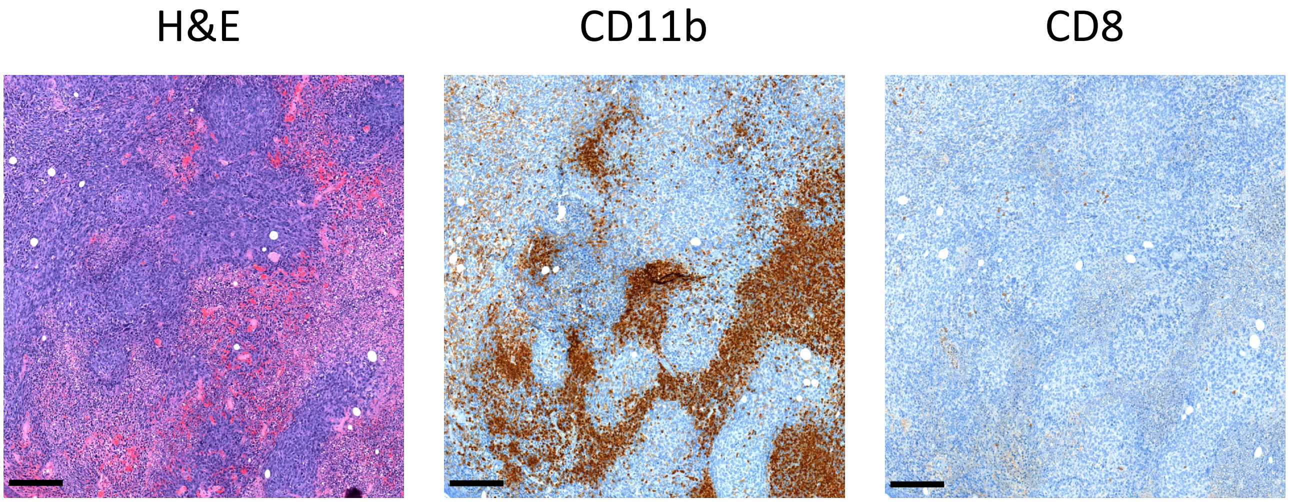

Figure S11: CD11b+ myeloid cells are confined to the stroma or necrotic areas of 4T1 tumors. 4T1 mouse breast cancer cells were injected and treated with RT, VTP or Cryoablation therapy according to the doses and schedule in Figure 1A. 6 days after treatment, tumors and spleens were harvested and processed for immunohistochemistry (IHC). Shown are representative images of serial sections for H&E, CD8 and CD11b for each treatment group. Bar = 200μm.

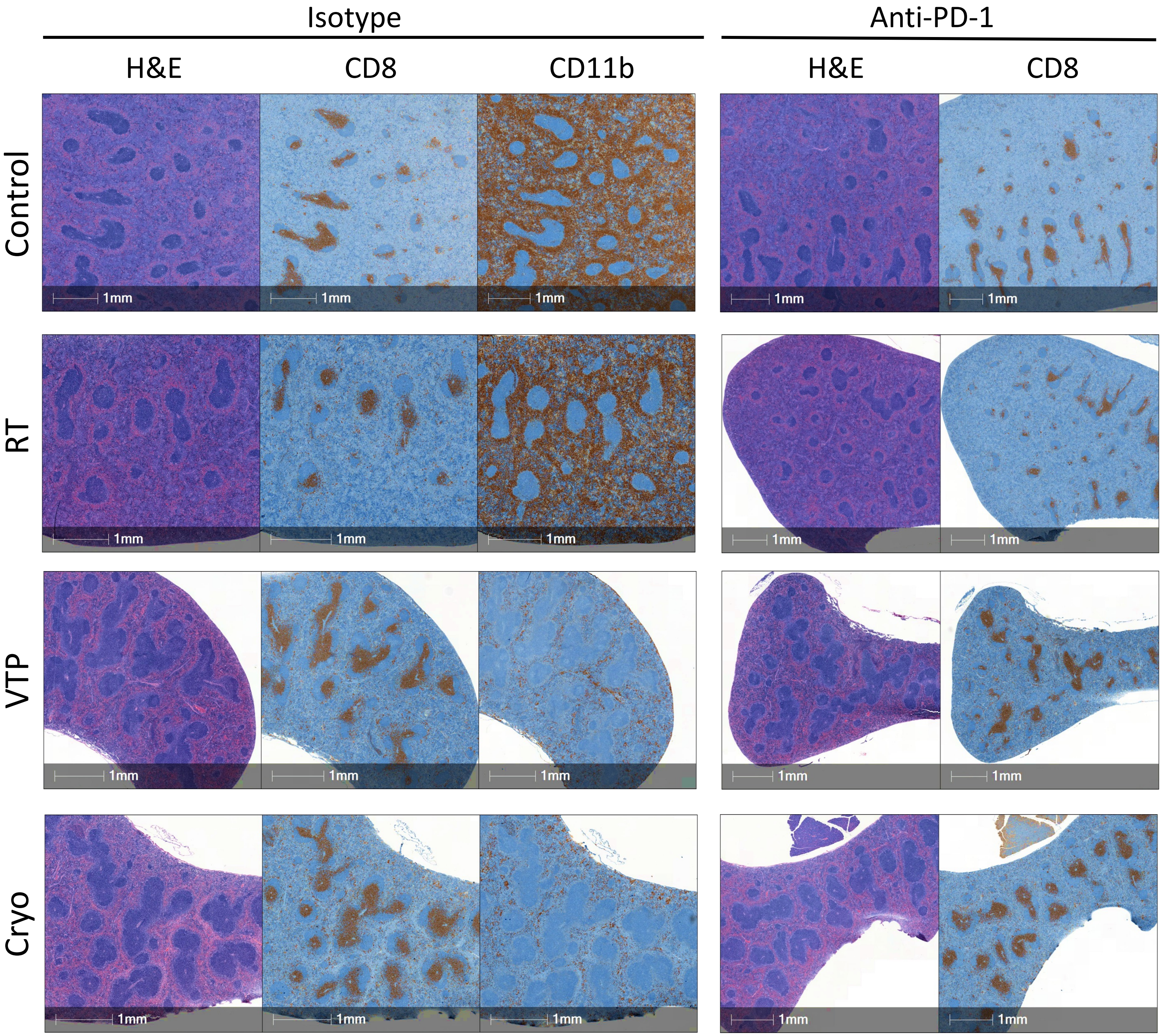

Figure S12: IHC analysis show a decrease in CD11b+ myeloid cells in the spleens of animals treated with VTP and Cryoablation. 10^5 4T1 mouse breast cancer cells were injected subcutaneously in the right hind limb of 6-8 week female Balbc mice and treated with RT, VTP or Cryo therapy according to the doses and schedule in Figure 1A. 6 days after treatment, spleens were harvested and processed for immunohistochemistry (IHC). Shown are representative images of serial sections for H&E, CD8 and CD11b for each treatment group. Bar = 1mm.
